# Electronic Nicotine Delivery System (ENDS) Device Types and Flavors Used by Youth in the PATH Study, 2016–2019

**DOI:** 10.3390/ijerph19095236

**Published:** 2022-04-26

**Authors:** Lisa D. Gardner, Sherry T. Liu, Haijun Xiao, Gabriella M. Anic, Karin A. Kasza, Eva Sharma, Andrew J. Hyland

**Affiliations:** 1 Office of Science, Center for Tobacco Products, US Food and Drug Administration, 10903 New Hampshire Ave, Silver Spring, MD 20993, USA; sherry.t.liu@fda.hhs.gov (S.T.L.); haijun.xiao@fda.hhs.gov (H.X.); gabriella.anic@fda.hhs.gov (G.M.A.); 2 Department of Health Behavior, Roswell Park Comprehensive Cancer Center, Buffalo, NY 14263, USA; karin.kasza@roswellpark.org (K.A.K.); andrew.hyland@roswellpark.org (A.J.H.); 3 Behavioral Health and Health Policy Group, Westat, 1600 Research Blvd, Rockville, MD 20850, USA; evasharma@westat.com

**Keywords:** adolescent, electronic nicotine delivery systems, tobacco products, population studies, flavors, regulation

## Abstract

The evolving electronic nicotine delivery system (ENDS) marketplace and recent regulatory actions may influence youth ENDS device preferences. Using data from Waves (W) 4, 4.5, and 5 (2016–2019) of the nationally representative Population Assessment of Tobacco and Health (PATH) Study, this study estimated the prevalence of open and closed system primary ENDS use by youth (12–17 years) current (past 30-day) ENDS users, and compared demographics, tobacco use characteristics, and patterns of ENDS use, including flavors, by device type. Among current ENDS users, closed system use was significantly higher than open system use in W4.5 (68.3% vs. 31.7%) and W5 (60.5% vs. 39.5%). In W5, closed system users were more likely to have a regular ENDS brand, believe their ENDS had nicotine, and use tobacco and mint or menthol flavors in the past 30 days compared to open system users. In W5, users of closed systems were less likely to use fruit, non-alcoholic drink, and candy, desserts, or other sweets flavors in the past 30 days than users of open systems. Youth were more likely to use closed over open system ENDS in 2017–2019. Differences were observed between device types, particularly with flavor use, reflecting recent changes in flavored product availability.

## 1. Introduction

Electronic nicotine delivery system (ENDS) use among U.S. youth has dramatically increased in the past decade [1,2]. Between 2011 and 2020, the prevalence of current (past 30-day) ENDS use increased from 1.5% to 19.6% in high school students and 0.6% to 4.7% in middle school students [1,3].

Since entering the U.S. marketplace in 2007, ENDS products have evolved to include a variety of device types such as cigalikes (products resembling cigarettes that may be rechargeable and refillable), disposables (non-refillable, non-rechargeable products that are not modifiable), tank-style (larger refillable, rechargeable products that include options for modifications), and cartridge/pod-based devices (prefilled, rechargeable products) [4,5]. Retail sales of prefilled cartridges substantially increased during 2014–2019, primarily driven by the introduction of JUUL and JUUL-like products [4,6,7]. Although retail sales of prefilled cartridges decreased and sales of disposable devices increased during 2019–2020, prefilled cartridges remained the leading type of ENDS product sold [4].

Use of flavored ENDS continues to be common among youth, with 82.9% of youth current ENDS users indicating use of a flavored product in 2020 [3]. During 2018–2019, significant changes in the availability of flavored products were observed in the marketplace. For example, JUUL suspended non-tobacco, non-menthol/non-mint-based flavored pod sales in brick-and-mortar retail stores in November 2018 and expanded this suspension online in October 2019 [8]. In November 2019, JUUL announced suspension of sales of mint-flavored pods in brick-and-mortar retail stores and online [9]. In January 2020, the Food and Drug Administration (FDA) released a guidance announcing the agency’s intention to prioritize enforcement against cartridge-based ENDS products without premarket authorization that come in flavors other than tobacco or menthol [10].

Research on U.S. youth surveyed between 2013 and 2016 prior to the widespread popularity of cartridge/pod-based devices during 2017–2018 suggested that youths used “open” system devices (rechargeable, refillable with e-liquid, may be modifiable) over “closed” system devices (disposable or use prefilled cartridges) [11,12,13,14]. Recent changes in the ENDS marketplace and tobacco regulatory actions may influence youth ENDS device preferences. In the 2019 National Youth Tobacco Survey, a majority of middle and high school students used closed system devices most often, primarily cartridge/pod-based devices [15]. Device characteristics such as device type may be associated with different user experiences, use patterns, flavor preferences, nicotine content and delivery, and user characteristics [14,16,17,18]; however, little is known about newer cartridge/pod-based devices. Understanding characteristics, the role of flavors, and patterns of use by open versus closed system users can help inform regulatory efforts to reduce youth ENDS use.

Using 2017–2019 data from the Population Assessment of Tobacco and Health (PATH) Study, a national longitudinal cohort study, this study provides more recent nationally representative estimates of youth current ENDS users by device type (closed and open systems), including newer cartridge/pod-based devices, and compares characteristics and patterns of use by device type. Additionally, this study assesses changes in ENDS device type prevalence and frequency of use among youth current ENDS users from 2016–2019.

## 2. Methods

### 2.1. Study Design and Population

The PATH Study is an ongoing, nationally representative, longitudinal cohort study of civilian, non-institutionalized U.S. adults (18+ years) and youth (12–17 years). Details regarding the PATH Study design and methods [19] and interview procedures, questionnaires, sampling, weighting, response rates, and accessing the data [20] are published elsewhere.

The study uses audio computer-assisted self-interviews (ACASI) available in English and Spanish to collect information on tobacco use patterns and associated health behaviors. Interviews were conducted with 14,798 youth at Wave (W) 4 (December 2016 to January 2018; although data collection ended on 3 January 2018, W4 is hereafter referred to as 2016–2017), 13,131 youth at W4.5 (December 2017 to November 2018), and 12,098 youth at W5 (December 2018 to November 2019). W4.5 was a special data collection limited to youth aged 12 to 17 at the time of the W4.5 interview.

The PATH Study employed a stratified address-based, area probability sampling design at W1 (September 2013 to December 2014). At W4, a probability replenishment sample of adults and youth was selected to account for loss of cohort members to follow-up, and was combined with the continuing sample (W1 respondents who were in the civilian, non-institutionalized population at W4) to form the W4 cohort. At W4, the weighted response rates for youth were 79.5% for the W4 continuing sample and 70.6% for the W4 cohort replenishment sample. For the W4 cohort (conditional upon participation at W4), the weighted response rates for youth were 89.1% at W4.5 and 83.5% at W5. The study was conducted by Westat and approved by the Westat Institutional Review Board. All youth respondents provided assent, while their parents/legal guardians provided informed consent.

At each wave, current ENDS use was defined as any ENDS use in the past 30 days, and was further categorized as either “not-light” (use of ENDS more than once in a lifetime) or “very-light” (use of ENDS only once in a lifetime) use. Analyses presented here focus on youth not-light current ENDS users exclusively. Sensitivity analyses including very-light ENDS users are presented as supplementary data.

### 2.2. ENDS Device Type

The PATH Study interview uses the term “electronic nicotine product” (ENP) to refer to ENDS devices. A respondent’s primary ENDS device was the ENP they used most often. In W4 and W4.5, Youth current not-light ENDS users were asked a series of binary questions to describe the ENP they used most often: (1) Is it rechargeable? (2) Does it use cartridges? (3) Can you refill it with “e-liquid”? Devices were then categorized as either closed systems (non-rechargeable devices or rechargeable devices that use cartridges) or open systems (rechargeable, refillable devices that do not use cartridges) [18,21,22].

A single-item approach to categorizing ENDS device types was proposed by Pearson et al. [23] to help standardize ENDS measures. In W5, youth current ENDS users were asked one question (“What kind of [ENP] is it?”) to categorize the ENP they used most often: “a disposable device” and “a device that uses replaceable prefilled cartridges” were closed systems; “a device with a tank that you refill with liquids” and “a mod system” were open systems.

Youth current ENDS users with missing data for device type (don’t know/refused; *n* = 14 for W4, *n* = 22 for W4.5, *n* = 11 for W5) or other device types (rechargeable, non-refillable devices that do not use cartridges for W4 [*n* = 20] and W4.5 [*n* = 19] and “something else” for W5 [*n* = 14]) were excluded from device type categorization. In W4 and W4.5, very-light ENDS users were not asked device type questions (*n* = 100 for W4, *n* = 104 for W4.5); in W5, they (*n* = 118) were excluded from device type categorization for comparability to earlier waves.

### 2.3. Demographics, Tobacco Use, and Characteristics and Patterns of ENDS Use

Demographics, tobacco use, and characteristics and patterns of ENDS use were categorized as shown in the tables. Tobacco use characteristics included current (any past 30-day) use of other combusted tobacco products (cigars, pipes, hookah, bidis, and kreteks) with and without cigarettes, current use of cigarettes, cigarette smoking status, cigarettes smoked per day (current smokers only), current use of non-combusted tobacco products (smokeless tobacco, snus, and dissolvables), exclusive ENDS use (current use of only ENDS, not any other tobacco products), and parents’ current use of any tobacco.

Respondents also reported characteristics and patterns of ENDS use, including having a regular brand of ENDS, ever use of ENDS fairly regularly, frequency of current ENDS use, belief that their ENDS contained nicotine, flavors used in the past 30 days, and reasons for ENDS use. Youth who knew the brand name of the ENDS they usually or last used were asked to provide it.

### 2.4. Statistical Analyses

Statistical analyses were conducted with SAS version 9.4 (SAS Institute, Cary, NC, USA) using full-sample and replicate weights (cross-sectional for W4, single-wave for W4.5 and W5) and the balanced repeated replication method [24] with Fay’s adjustment of 0.3 to account for the PATH Study’s complex sample design and increase estimate stability [25].

**Figure 1:**
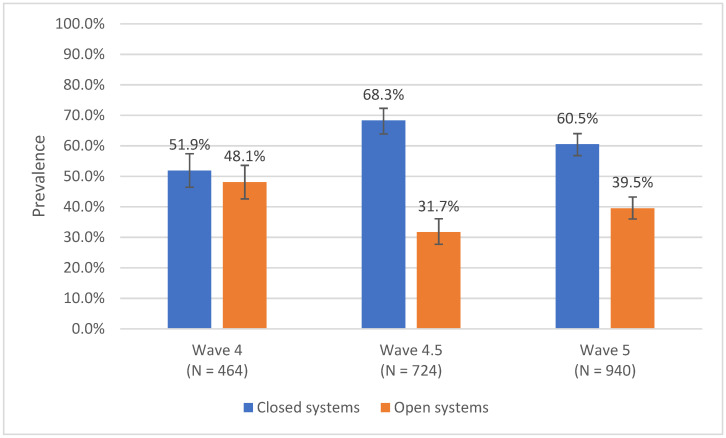
Prevalence of closed and open systems in youth not-light current ENDS users, PATH Study Waves 4, 4.5, and 5 ^a,b^. Compared to open system use, the odds of closed system use for each one wave increase was 1.11 times (95% CI: 0.97, 1.28) the odds of closed system use at the previous wave (*p* = 0.132) ^c^. ^a^ Current ENDS use is any ENDS use in the past 30 days. Not-light current ENDS users used ENDS more than once in their lifetime. For device type—Waves 4 and 4.5: Closed systems are devices that are not rechargeable, or devices that are rechargeable and use cartridges; open systems are devices that are rechargeable, do not use cartridges, and are refillable. Wave 5: Closed systems are disposable e-cigarettes or e-cigarettes that use pre-filled pods or cartridges; open systems are e-cigarettes with a refillable tank or mod systems. Device type categorization for Wave 5 includes not-light current ENDS users only (i.e., the primary analytic population). ^b^ Wave 4 is 2016–2017, Wave 4.5 is 2017–2018, and Wave 5 is 2018–2019. For each wave, percentage estimates of the prevalence of primary closed and open system ENDS use are weighted. Ns presented are unweighted. ^c^ Unadjusted logistic regression was used to test for a trend (cross-sectional) in the prevalence of primary closed system ENDS use (compared to open system) across waves. Abbreviations: ENDS = Electronic nicotine delivery system; PATH = Population Assessment of Tobacco and Health.

The prevalence of open and closed system primary ENDS devices in all youth and in youth current ENDS users with known device type (with 95% confidence intervals (CIs)) was estimated in W4, W4.5, and W5 (Table 1). Differences in the proportion of open and closed systems at each wave were evaluated using Rao-Scott chi-square tests. We also used unadjusted logistic regression to test for trends in the prevalence of closed systems (compared to open systems) (Figure 1) and the frequency of current ENDS use (overall and by device type) across waves (Figures S1–S3).

Cross-sectional estimates (weighted percentages with 95% CI:s) of demographics, tobacco use characteristics, device characteristics, and patterns of ENDS use were examined by primary device type (closed vs. open systems) in W4.5 and W5 (Tables S1–S4), to focus on data collected after the widespread popularity of cartridge/pod-based devices. As this proliferation occurred during W4 data collection (2016–2017) [4,6,7], W4 data was not included for comparison. Unadjusted logistic regression was used to assess differences between primary device types by comparing the odds of closed vs. open system use for each variable.

Sensitivity analyses were completed to determine whether adding very-light current ENDS users changed estimates for W5 (Tables S5 and S6). We also compared demographics and tobacco use characteristics for not-light vs. very-light ENDS users in W4.5 and W5, using unadjusted logistic regression, to further characterize very-light users (Tables S7 and S8).

## 3. Results

### 3.1. ENDS Device Type

In all youth, the prevalence of current ENDS use was 4.3% (95% CI: 3.9, 4.8) in W4 (2016–2017), 6.8% (95% CI: 6.3, 7.3) in W4.5 (2017–2018), and 8.6% (95% CI: 8.1, 9.1) in W5 (2018–2019) (Table 1). Among current ENDS users, the prevalence of closed and open systems was 51.9% vs. 48.1% in W4, 68.3% vs. 31.7% in W4.5, and 60.5% vs. 39.5% in W5. There was a non-significant positive trend in the odds of closed vs. open systems across three waves (Figure 1).

In W5, primary ENDS devices were 4.3% disposable, 56.1% replaceable prefilled cartridges, 33.3% tanks with refillable liquids, and 6.3% mod systems (Table 1).

### 3.2. Demographics and Tobacco Use Characteristics

There were significant differences in demographics and tobacco use between open and closed system users in both waves (Tables S1 and S2). Compared to youth who got mostly A’s, the odds of closed system use were significantly lower in those who received mostly B’s (W4.5: OR = 0.61; W5: OR = 0.67), C’s (W4.5: OR = 0.56; W5: OR = 0.64), and D’s or lower (W4.5 only: OR = 0.34). The odds of closed system use were also lower in youth whose parents currently used any (vs. no) tobacco (W4.5: OR = 0.54, 95% CI: 0.38, 0.78; W5: OR = 0.62, 95% CI: 0.47, 0.82).

Unlike W4.5, older youth were more likely to use closed than open systems in W5. The odds of closed system use in 16- to 17-year-olds in W5 was 2.30 times (95% CI: 1.01, 5.24) the odds in 12- to 13-year-olds. Closed system use was also more likely in 9–12th grade vs. 6–8th grade (OR = 2.09, 95% CI: 1.22, 3.61). Additionally, in W5 only, current smokers who smoked 20–30 days in the past month were less likely to use closed systems compared to never smokers (OR = 0.43, 95% CI: 0.22, 0.84).

### 3.3. Characteristics and Patterns of ENDS Use

Characteristics and patterns of ENDS use also varied between open and closed system users at each wave (Tables S3 and S4). At both waves, youth who believed their ENDS contained nicotine (W4.5: OR = 2.90, 95% CI: 1.74, 4.82; W5: OR = 3.21, 95% CI: 2.23, 4.62) or didn’t know (W4.5: OR = 2.83, 95% CI: 1.69, 4.73; W5: OR = 2.37, 95% CI: 1.34, 4.20) had higher odds of closed system use than those who did not. In youth who had a regular brand of ENDS (W4.5: OR = 2.40, 95% CI: 1.48, 3.89; W5: OR = 4.49, 95% CI: 3.15, 6.39) or did not know (W5 only: OR = 1.66, 95% CI: 1.04, 2.64), the odds of closed system use were higher than the odds in youth without a regular brand. In W4.5 only, youth who used their ENDS device more frequently (20–30 days in the past month) had lower odds of closed system use (OR = 0.70, 95% CI: 0.50, 0.98) than less frequent ENDS users (1–19 days). Across all three waves, there was a significant positive trend in the odds of more frequent (20–30 days) ENDS use, overall (OR = 1.34, 95% CI: 1.16, 1.56; Figure S1) and in closed system users (OR = 1.49, 95% CI: 1.21, 1.84; Figure S3).

Compared to open systems, youth in both waves who used closed systems were more likely to use mint or menthol (W4.5: OR = 3.26, 95% CI: 2.23, 4.76; W5: OR = 2.30, 95% CI: 1.71, 3.09) and tobacco flavor (W4.5: OR = 4.04, 95% CI: 1.79, 9.12; W5: OR = 1.67, 95% CI: 1.04, 2.66) as flavors in the past 30 days and less likely to use candy, dessert, or other sweet flavors (W4.5: OR = 0.59, 95% CI: 0.43, 0.81; W5: OR = 0.39, 95% CI: 0.28, 0.53). Although tobacco flavor use differed between closed (14.7%) and open (4.1%) systems in W4.5, there was low precision for open system users. In W5 only, youth who used closed systems were also less likely to use fruit (OR = 0.52, 95% CI: 0.37, 0.73) and non-alcoholic drink (OR = 0.32, 95% CI: 0.18, 0.55) flavors. In W5, flavors used most often in the past 30 days were mint or menthol (42.1% closed, 18.1% open), fruit (41.6% closed, 55.2% open), and candy, desserts, or other sweets (10.3% closed, 18.4% open).

At both waves, users of closed systems were less likely to endorse “E-liquid comes in flavors I like” as a reason for ENDS use (W4.5: OR = 0.68, 95% CI: 0.47, 0.98; W5: OR = 0.61, 95% CI: 0.45, 0.82). Closed system users were less likely to endorse “They might be less harmful to people around me than cigarettes” (OR = 0.68, 95% CI: 0.48, 0.96) as a reason for ENDS use in W4.5, and “They are affordable” (OR = 0.63, 95% CI: 0.45, 0.88) in W5.

### 3.4. ENDS Brands by Device Type

Overall, 27.9% in W4.5 and 40.5% in W5 knew the brand name of the ENDS they usually or last used, and JUUL, Smok, Blu Cigs, and E-Swisher were the top brands used in both waves. For closed systems, the most used ENDS brand was JUUL (W4.5: 47.4%, 95% CI: 37.2, 57.8; W5: 77.2%, 95% CI: 71.9, 81.8). For open systems, the most commonly reported ENDS brand used was Smok in W4.5 (42.6%, 95% CI: 27.7, 59.0) and JUUL in W5 (26.9%, 95% CI: 18.2, 37.9).

## 4. Discussion

Research prior to the introduction of cartridge/pod-based devices found that youth were more likely to use open systems that were rechargeable, refillable, and/or modifiable over closed systems such as disposables and cigalikes [11,12,13,14]. These PATH Study data suggest that more youth ENDS users used closed systems over open systems during 2017–2018 (W4.5: 68.3% vs. 31.7%) and 2018–2019 (W5: 60.5% vs. 39.5%), consistent with more recent research suggesting that cartridge/pod-based devices are more common among youth [15,26,27]. In our study, few youth ENDS users in 2018–2019 used a disposable device most often; more than 9 in 10 closed system users used a device with replaceable prefilled cartridges.

Findings from this study suggest key differences in the characteristics and patterns of use between open and closed system youth users. For instance, in the most recent wave of data (2018–2019), closed system users were less likely to have parents who currently used any tobacco, but were more likely to be in 9–12th grade, achieve better grades in school, have a regular brand of ENDS, and believe their ENDS contained nicotine. Compared to open system users, closed system users in 2018–2019 were less likely to endorse “E-liquid comes in flavors I like” and “They are affordable” as reasons for ENDS use.

From 2016–2019, there were significant increases in more frequent ENDS use overall and in closed system users, consistent with the idea that closed systems containing nicotine salts deliver higher levels of nicotine and may lead to increased dependence among youth [28,29]. No differences in the frequency of current ENDS use were observed between closed and open systems users in 2018–2019. Frequent ENDS use has been associated with youth use of open refillable system devices in prior studies [11,12], including in earlier waves of the PATH Study [11], but definitions of frequent ENDS use varied by study.

Overall use of flavors was high from 2017–2019, with fruit and mint/menthol as the two most used flavors. However, mint/menthol flavor use increased from 2017–2018 (44.5%) to 2018–2019 (57.3%), while use of other flavors (e.g., fruit) decreased or stayed the same. Mint and menthol were combined in the response option and could not be assessed separately. During 2018–2019, closed system users were more likely to use tobacco or mint/menthol flavors in the past 30 days compared to open system users, who were more likely to use fruit, non-alcoholic drink, and candy, desserts, or other sweet flavors. This is consistent with prior research in youth and adult ENDS users [14,18]. Marketplace changes during 2018–2019 in the availability of flavored products (e.g., JUUL’s suspension of non-tobacco, non-menthol/non-mint flavored pod sales) may help explain flavor preferences of ENDS users in W5 [8,9]. In January 2020, the FDA prioritized the enforcement of flavored, cartridge-based ENDS products (other than tobacco- or menthol-flavored) without premarket authorization; disposable ENDS products were not subject to this enforcement prioritization at that time [10]. Data from our study demonstrate what ENDS devices youth were using before the FDA’s enforcement prioritization. In the future, PATH Study data can inform monitoring the impact of the enforcement prioritization on device types and flavors used among youth.

We note several limitations. First, device type misclassification was possible based on inconsistencies in how open and closed systems were classified in both waves. For example, in W5, JUUL was the most used “open” brand, despite being a closed system. Furthermore, in earlier studies using PATH Study data, all refillable devices were considered open systems [11,14], whereas the way device types were categorized in this study, closed systems could also be refillable. In our study, over 70% of closed systems were refillable in W4 (78.1%) and W4.5 (72.8%). This may reflect a distinction between how these devices are supposed to be used and how they are used in practice. In the 2019 FDA guidance, “Premarket Tobacco Product Applications for Electronic Nicotine Delivery Systems,” closed ENDS are defined as devices that are not refillable or that use “e-liquid contained in replaceable cartridges or pods that are not intended to be refillable” [30]. There are reports of refilling closed pods that are not meant to be refilled [31,32], although it is not clear how common this practice is. Ambiguity and changes in ENDS terminology may have also contributed to misclassification. In W5, youth may have had difficulty categorizing the ENDS product they use most often, particularly products that may be refillable pod-based systems. Over the past few years, the rapidly evolving ENDS marketplace and the substantial diversity across ENDS products have made device categorization difficult [5,22], and research must continually evaluate whether the significance of device type as a marker of appeal, addiction, and toxicity has changed, as well as whether sufficiently meaningful differences in ENDS user characteristics and use patterns by device type exist for policy and regulation purposes. Second, the PATH Study interview changed in terms of how device type was assessed across waves to reflect changes in the marketplace. In W4 and W4.5, a series of binary questions were used to categorize primary device type. In W5, a single item was used to determine primary device type. The study population asked about device type was also expanded in W5 to include very-light current ENDS users; in our sensitivity analyses, although very-light users differed from not-light users on several characteristics at W4.5 and W5 (Tables S7 and S8), the addition of very-light users in W5 resulted in only minor changes by device type (Tables S5 and S6). These differences in interview questions, although reflective of the changing marketplace, make it difficult to assess changes in closed vs. open system use prevalence over time. Third, due to the cross-sectional nature of this analysis, assessment of causality between device type and characteristics or use patterns is not possible. Lastly, this study only examines the most often used ENDS device and may underestimate prevalence of each device type; Krishnan-Sarin et al. [27] found that two-thirds of youth reported using multiple device types.

## 5. Conclusions

In summary, this study provides recent nationally representative estimates of youth current ENDS use by device type (open vs. closed systems) using data from the PATH Study, a longitudinal cohort study. It also provides pre-enforcement data that demonstrate what ENDS devices youth were using prior to the FDA’s 2020 enforcement prioritization [10]. In 2017–2019, youth current ENDS users most often used closed system devices. Differences in characteristics and use patterns were observed between closed and open system users, including reasons for ENDS use and use of flavors. This analysis lays the foundational work for understanding device types and flavors used among youth that can be further examined using longitudinal PATH Study data. Given the increasing prevalence of current ENDS use in youth, a better understanding of the device types that youth use, as well as the characteristics associated with their use, remains a priority.

## Figures and Tables

**Table 1:** Prevalence of youth current ENDS use, overall and by device type, PATH Study Waves 4, 4.5, and 5 ^a^.

	Unweighted N	Overall Prevalence Weighted % (95% CI:)	Prevalence in Youth Not-Light Current ENDS Users with Known Device Type ^b^ Weighted % (95% CI:)	*p*-Value ^c^ (Rao-Scott χ^2^)
**Wave 4**	14,793 ^d^			
Current use of ENDS ^e^	598	4.3 (3.9, 4.8)	–	
Closed systems	238	1.7 (1.5, 2.1)	51.9 (46.4, 57.4)	0.490
Open systems	226	1.6 (1.4, 1.9)	48.1 (42.6, 53.6)	
**Wave 4.5**	12,918 ^d^			
Current use of ENDS ^e^	869	6.8 (6.3, 7.3)	–	
Closed systems	485	3.9 (3.5, 4.3)	68.3 (63.9, 72.3)	<0.001
Open systems	239	1.8 (1.6, 2.1)	31.7 (27.7, 36.1)	
**Wave 5**	11,976 ^d^			
Current use of ENDS ^e^	1083	8.6 (8.1, 9.1)	–	
Closed systems	549	4.5 (4.1, 4.9)	60.5 (56.8, 64.0)	<0.001
A disposable device	40	0.3 (0.2, 0.5)	4.3 (3.1, 6.0)	
A device that uses replaceable prefilled cartridges	509	4.2 (3.8, 4.6)	56.1 (52.4, 59.9)	
Open systems	391	3.0 (2.7, 3.3)	39.5 (36.0, 43.2)	
A device with a tank that you refill with liquids	328	2.5 (2.2, 2.8)	33.3 (30.0, 36.7)	
A mod system	63	0.5 (0.4, 0.6)	6.3 (4.8, 8.1)	

^a^ Current ENDS use is any ENDS use in the past 30 days. Not-light current ENDS users used ENDS more than once in their lifetime. For device type—Waves 4 and 4.5: Closed systems are devices that are not rechargeable, or devices that are rechargeable and use cartridges; open systems are devices that are rechargeable, do not use cartridges, and are refillable. Wave 5: Closed systems are disposable e-cigarettes or e-cigarettes that use pre-filled pods or cartridges; open systems are e-cigarettes with a refillable tank or mod systems. ^b^ Device type categorization includes not-light current ENDS users only (i.e., the primary analytic population). Very-light current ENDS users used ENDS only once in their lifetime. In Waves 4 and 4.5, very-light current ENDS users were not asked device type questions. In Wave 5, the device type question was asked of both very-light and not-light current ENDS users. Very-light current ENDS users (*n* = 118) in Wave 5 were excluded from the primary analyses of current ENDS users for comparability to earlier waves. ^c^ The one-way Rao-Scott chi-square is a goodness of fit test for the null hypothesis of equal proportions of open and closed systems in non-light current ENDS users with known device type at each wave. ^d^ Differences in the total youth interviewed at each wave and the sample sizes provided here are due to missing cross-sectional or single-wave weights at Wave 4 (*n* = 5), Wave 4.5 (*n* = 213), and Wave 5 (*n* = 122). Cross-sectional weights (Wave 4) were missing for youth in the continuing sample who were not in the civilian, non-institutionalized population at Wave 4. Single-wave weights (Waves 4.5 and 5) were missing for youth who responded at Wave 4, but not at Wave 4.5 or Wave 5, respectively. ^e^ Includes current ENDS users who were excluded from device type categorization: very-light ENDS users, and don’t know or refused and “other” responses. In Waves 4 and 4.5, “other” devices were rechargeable, did not use cartridges, but were not refillable. In Wave 5, the “other” response option was listed as “something else” and respondents were asked to specify a write-in response. In current ENDS users, the prevalence of those excluded from device type categorization was 22.5% (95% CI: 19.0, 26.4; *n* = 134) in Wave 4, 16.0% (95% CI: 13.7, 18.7; *n* = 145) in Wave 4.5, and 12.7% (95% CI: 10.8, 14.8; *n* = 143) in Wave 5. Abbreviations: CI = confidence interval; ENDS = electronic nicotine delivery system; PATH = Population Assessment of Tobacco and Health.

## Data Availability

Details on accessing the PATH Study data are described in the *PATH Study Restricted Use Files* website located at https://doi.org/10.3886/ICPSR36231.v29 (accessed on 18 February 2022). Access to these data is restricted. Users interested in accessing these data must apply for access and complete a Restricted Data Use Agreement.

## Supplementary Materials

The following supporting information can be downloaded at: https://www.mdpi.com/article/10.3390/ijerph19095236/s1. All supplementary tables and figures are listed below: Table S1. Demographic and Tobacco Use Characteristics of Youth Current ENDS Users by Device Type, PATH Study Wave 4.5. Table S2. Demographic and Tobacco Use Characteristics of Youth Current ENDS Users by Device Type, PATH Study Wave 5. Table S3. Characteristics and Patterns of ENDS Use Among Youth Current ENDS Users by Device Type, PATH Study Wave 4.5. Table S4. Characteristics and Patterns of ENDS Use Among Youth Current ENDS Users by Device Type, PATH Study Wave 5. Table S5. Demographic and Tobacco Use Characteristics of Youth Current ENDS Users by Device Type (Not-Light and Very-Light ENDS Users), PATH Study Wave 5. Table S6. Characteristics and Patterns of ENDS Use Among Youth Current ENDS Users by Device Type (Not-Light and Very-Light ENDS Users), PATH Study Wave 5. Table S7. Demographic and Tobacco Use Characteristics of Youth Current ENDS Users by Not-Light vs. Very-Light ENDS Use, PATH Study Wave 4.5. Table S8. Demographic and Tobacco Use Characteristics of Youth Current ENDS Users by Not-Light vs. Very-Light ENDS Use, PATH Study Wave 5. Figure S1. Frequency (1–19 vs. 20–30 Days) of Current ENDS Use in Youth Not-Light Current ENDS Users—Overall, PATH Study Waves 4, 4.5, and 5. Figure S2. Frequency (1–19 vs. 20–30 Days) of Current ENDS Use in Youth Not-Light Current ENDS Users—Open Systems, PATH Study Waves 4, 4.5, and 5. Figure S3. Frequency (1–19 vs. 20–30 Days) of Current ENDS Use in Youth Not-Light Current ENDS Users–Closed Systems, PATH Study Waves 4, 4.5, and 5.
