# Supplementary material for: Electronic Nicotine Delivery System (ENDS) Device Types and Flavors Used by Youth in the PATH Study, 2016–2019
Source: Int J Environ Res Public Health. 2022 Apr 26;19(9):5236. doi: 10.3390/ijerph19095236 (PMC9101111; doi:10.3390/ijerph19095236)
Supplement: Supplementary file 1 [file ijerph-19-05236-s001.zip › ijerph-1624652-supplementary.pdf]

Table S1. Demographic and Tobacco Use Characteristics of Youth Current ENDS Users by Device Type, PATH Study Wave 4.5.[a]

	Overall (n = 724)	Closed Systems (n = 485)	Open Systems (n = 239)	OR[b]	95% CI
	**Weighted % (95% CI)**	**Weighted % (95% CI)**	**Weighted % (95% CI)**		
**Sex**					
Male	52.1 (48.2, 56.0)	52.0 (47.2, 56.8)	52.3 (45.2, 59.3)	REF	REF
Female	47.9 (44.0, 51.8)	48.0 (43.2, 52.8)	47.7 (40.7, 54.8)	1.01	(0.71, 1.44)
**Age (years)**					
12-13	4.1 (2.7, 6.2)	4.1 (2.4, 7.2)	4.1 (2.2, 7.8)†	REF	REF
14-15	30.8 (27.5, 34.2)	30.3 (26.4, 34.5)	31.8 (25.6, 38.7)	0.95	(0.34, 2.68)
16-17	65.1 (61.4, 68.5)	65.5 (60.9, 69.9)	64.1 (56.9, 70.7)	1.02	(0.36, 2.88)
**Race/ethnicity**					
White, non-Hispanic	69.8 (66.2, 73.0)	70.1 (65.7, 74.1)	69.1 (62.7, 74.8)	REF	REF
Black, non-Hispanic	3.8 (2.6, 5.6)	3.8 (2.4, 6.0)	3.9 (2.0, 7.2)†	0.97	(0.40, 2.37)
Other, non-Hispanic[c]	8.0 (6.2, 10.2)	8.6 (6.1, 11.9)	6.7 (4.1, 10.7)	1.26	(0.63, 2.53)
Hispanic	18.5 (15.8, 21.5)	17.6 (14.4, 21.3)	20.4 (15.7, 26.0)	0.85	(0.55, 1.32)
**Current grade level[d]**					
6-8th grade	5.3 (3.7, 7.5)	4.8 (2.8, 7.9)	6.5 (3.9, 10.6)	REF	REF
9-12th grade	91.1 (88.2, 93.3)	92.0 (88.7, 94.4)	89.1 (83.3, 93.0)	1.41	(0.60, 3.32)
**Grades[e]**					
Mostly A's or A's and B's	51.9 (47.5, 56.1)	56.4 (51.3, 61.4)	42.0 (35.2, 49.0)	REF	REF
Mostly B's or B's and C's	29.8 (26.4, 33.5)	27.8 (23.6, 32.5)	34.2 (28.4, 40.5)	**0.61***	(0.41, 0.89)
Mostly C's or C's and D's	13.8 (11.1, 17.0)	12.5 (9.4, 16.4)	16.6 (12.2, 22.3)	**0.56***	(0.33, 0.95)
Mostly D's, D's and F's, or mostly F's	4.5 (3.2, 6.3)	3.3 (2.0, 5.3)	7.2 (4.5, 11.3)	**0.34****	(0.16, 0.70)
**Current use of other combusted products[f]**	26.1 (22.9, 29.6)	25.3 (21.3, 29.7)	28.0 (22.6, 34.0)	0.87	(0.61, 1.25)
**Current use of cigarettes**	21.4 (18.4, 24.8)	20.3 (16.9, 24.2)	23.7 (18.8, 29.5)	0.82	(0.58, 1.17)

**Cigarette smoking status**					
Never smoker	51.8 (47.6, 56.0)	52.5 (47.4, 57.5)	50.4 (44.1, 56.7)	REF	REF
Ever smoker, but not within past month	26.9 (23.4, 30.8)	27.5 (23.4, 32.1)	25.7 (20.4, 31.7)	1.03	(0.72, 1.47)
Current smoker, 1-19 days during past month	17.2 (14.6, 20.1)	16.5 (13.4, 20.1)	18.6 (13.9, 24.5)	0.85	(0.54, 1.34)
Current smoker, 20-30 days during past month	4.1 (2.8, 5.9)	3.5 (2.2, 5.6)	5.3 (3.1, 8.9)	0.64	(0.34, 1.24)
**Cigarettes smoked per day (current smokers only)**[g]					
<1	35.0 (27.6, 43.2)	36.9 (27.4, 47.6)	31.5 (19.6, 46.5)	REF	REF
1-5	54.8 (46.1, 63.2)	52.6 (41.3, 63.7)	58.6 (44.4, 71.5)	0.77	(0.32, 1.84)
6+	10.2 (6.4, 16.0)	10.4 (5.8, 17.9)	9.9 (4.4, 20.7)†	0.90	(0.23, 3.59)
**Current use of non-cigarette combusted products**[h]	10.8 (8.7, 13.2)	11.3 (8.7, 14.6)	9.5 (6.4, 13.9)	1.22	(0.73, 2.04)
**Current use of non-combusted products**[i]	6.1 (4.5, 8.4)	6.2 (4.1, 9.2)	6.0 (3.6, 9.8)	1.03	(0.55, 1.94)
**Exclusive ENDS use**[j]	70.6 (67.2, 73.9)	71.4 (66.8, 75.5)	69.1 (62.9, 74.7)	1.12	(0.76, 1.64)
**Parents' current use of any tobacco**[k]	28.5 (24.4, 32.9)	24.4 (20.3, 29.0)	37.3 (30.3, 45.0)	**0.54****	(0.38, 0.78)

[a] Analyses are limited to youth not-light current ENDS users. Current ENDS use is any ENDS use in the past 30 days. Not-light current ENDS users used ENDS more than once in their lifetime. For device type – Closed systems are devices that are not rechargeable, or devices that are rechargeable and use cartridges; open systems are devices that are rechargeable, do not use cartridges, and are refillable.

[b] The unadjusted odds ratio is the odds of closed vs. open system use for each level of a variable compared to the reference level for that variable (noted explicitly as REF or implicitly as "no" for binary yes/no variables).

[†] Estimate should be interpreted with caution because it has low precision. It is based on a denominator sample size of less than 50, or the coefficient of variation of the estimate or its complement is larger than 30%.

[c] Includes Asian, American Indian or Alaska Native, Native Hawaiian, Guamanian or Chamorro, Samoan, other Pacific Islander, and respondents who selected non-Hispanic multiple races.

[d] Current grade level was asked of youth who went to school in the past 12 months. Respondents who selected 5th grade and lower, college, vocational, or technical school, not enrolled, home-schooled, and ungraded were categorized as other (n = 26; data not shown).

[e] Grades were reported by youths' parents or guardians and reflect performance at school in the past 12 months. Excludes ungraded school.

[f] Combusted tobacco products included cigarettes, cigars, pipes, hookah, bidis, and kreteks.

[g] Cigarettes smoked per day was asked of current cigarette smokers.

[h] Non-cigarette combusted tobacco products included cigars, pipes, hookah, bidis, and kreteks.

[i] Non-combusted tobacco products included smokeless tobacco, snus, and dissolvables.

[j] Exclusive ENDS use included respondents who only used ENDS in the past 30 days, not any other tobacco products (combusted or non-combusted).

[k] Current use of any tobacco was reported by youths' parents or guardians, and excludes those who already completed a parent interview for another youth. Includes any tobacco use from parents in the past 30 days, including cigarettes, traditional cigars, cigarillos, filtered cigars, pipe, hookah, snus, smokeless tobacco, ENDS, and dissolvable tobacco.

* p <0.05, ** p <0.01, *** p <0.001.

Abbreviations: CI = confidence interval; ENDS = electronic nicotine delivery system; OR = unadjusted odds ratio; PATH = Population Assessment of Tobacco and Health; REF = reference level.

Table S2. Demographic and Tobacco Use Characteristics of Youth Current ENDS Users by Device Type, PATH Study Wave 5.[a]

	Overall (n = 940)	Closed Systems (n = 549)	Open Systems (n = 391)	OR[b]	95% CI
	Weighted % (95% CI)	Weighted % (95% CI)	Weighted % (95% CI)		
**Sex**					
Male	50.9 (47.7, 54.1)	50.2 (45.6, 54.8)	52.0 (46.3, 57.7)	REF	REF
Female	49.1 (45.9, 52.3)	49.8 (45.2, 54.4)	48.0 (42.3, 53.7)	1.08	(0.78, 1.49)
**Age (years)**					
12-13	3.9 (2.8, 5.4)	2.7 (1.5, 4.8)	5.6 (3.7, 8.5)	REF	REF
14-15	30.3 (27.1, 33.6)	28.8 (24.6, 33.3)	32.6 (27.2, 38.4)	1.83	(0.77, 4.33)
16-17	65.9 (62.5, 69.1)	68.5 (63.8, 72.9)	61.8 (56.1, 67.2)	**2.30***	(1.01, 5.24)
**Race/ethnicity**					
White, non-Hispanic	69.5 (66.2, 72.5)	71.0 (66.7, 75.0)	67.0 (62.1, 71.6)		
Black, non-Hispanic	5.1 (3.8, 6.8)	4.5 (3.0, 6.8)	6.1 (4.0, 9.1)	0.70	(0.37, 1.32)
Other, non-Hispanic[c]	7.8 (6.2, 9.8)	8.6 (6.3, 11.8)	6.5 (4.4, 9.4)	1.26	(0.79, 2.03)
Hispanic	17.6 (15.2, 20.3)	15.8 (13.0, 19.2)	20.4 (16.6, 24.8)	0.73	(0.52, 1.02)
**Current grade level[d]**					
6-8th grade	7.5 (5.7, 9.7)	5.3 (3.7, 7.6)	10.8 (7.5, 15.3)	REF	REF
9-12th grade	87.8 (85.4, 89.9)	89.1 (86.0, 91.5)	85.8 (81.3, 89.4)	**2.09****	(1.22, 3.61)
**Grades[e]**					
Mostly A's or A's and B's	49.6 (46.1, 53.1)	53.9 (49.5, 58.2)	43.1 (37.9, 48.5)	REF	REF
Mostly B's or B's and C's	29.9 (27.1, 33.0)	27.8 (23.8, 32.1)	33.3 (28.7, 38.2)	**0.67***	(0.48, 0.94)
Mostly C's or C's and D's	14.5 (12.3, 16.9)	13.2 (10.5, 16.4)	16.4 (12.8, 20.8)	**0.64***	(0.43, 0.97)
Mostly D's, D's and F's, or mostly F's	6.0 (4.5, 8.0)	5.2 (3.6, 7.5)	7.2 (4.8, 10.6)	0.58	(0.33, 1.02)
**Current use of other combusted products[f]**	22.1 (19.0, 25.6)	20.1 (16.5, 24.2)	25.3 (20.6, 30.7)	0.74	(0.53, 1.04)
**Current use of cigarettes**	18.4 (15.4, 21.9)	16.1 (12.9, 20.0)	21.9 (17.2, 27.6)	0.68	(0.47, 1.00)

**Cigarette smoking status**					
Never smoker	55.1 (51.9, 58.3)	57.7 (53.6, 61.8)	51.1 (46.0, 56.2)	REF	REF
Ever smoker, but not within past month	26.5 (23.8, 29.4)	26.1 (22.6, 30.0)	27.2 (23.0, 31.8)	0.85	(0.63, 1.15)
Current smoker, 1-19 days during past month	14.3 (11.7, 17.2)	13.3 (10.3, 17.0)	15.8 (12.0, 20.4)	0.75	(0.48, 1.16)
Current smoker, 20-30 days during past month	4.1 (3.0, 5.6)	2.9 (1.8, 4.6)	6.0 (3.9, 8.9)	**0.43***	(0.22, 0.84)
**Cigarettes smoked per day (current smokers only)[g]**					
<1	41.7 (34.7, 49.1)	48.7 (38.7, 58.9)	33.8 (23.8, 45.6)	REF	REF
1-5	43.5 (35.0, 52.4)	41.1 (31.5, 51.4)	46.2 (34.4, 58.4)	0.62	(0.31, 1.24)
6+	14.8 (10.2, 21.0)	10.2 (5.0, 19.7)†	20.0 (12.3, 30.8)	0.35	(0.10, 1.21)
**Current use of non-cigarette combusted products[h]**	7.8 (6.2, 9.8)	7.1 (5.0, 10.0)	8.8 (6.3, 12.0)	0.80	(0.49, 1.31)
**Current use of non-combusted products[i]**	4.3 (3.2, 5.9)	5.0 (3.5, 7.2)	3.3 (2.0, 5.6)	1.52	(0.85, 2.71)
**Exclusive ENDS use[j]**	74.8 (71.4, 77.9)	76.0 (71.8, 79.8)	72.8 (67.5, 77.5)	1.19	(0.86, 1.64)
**Parents' current use of any tobacco[k]**	36.1 (32.4, 39.9)	31.7 (27.5, 36.1)	42.8 (37.3, 48.5)	**0.62*****	(0.47, 0.82)

[a] Analyses are limited to youth not-light current ENDS users. Current ENDS use is any ENDS use in the past 30 days. Not-light current ENDS users used ENDS more than once in their lifetime. For device type – Closed systems are disposable e-cigarettes or e-cigarettes that uses pre-filled pods or cartridges; open systems are e-cigarettes with a refillable tank or mod systems. Device type categorization for Wave 5 includes not-light current ENDS users only (i.e., the primary analytic population).

[b] The unadjusted odds ratio is the odds of closed vs. open system use for each level of a variable compared to the reference level for that variable (noted explicitly as REF or implicitly as "no" for binary yes/no variables).

[†] Estimate should be interpreted with caution because it has low precision. It is based on a denominator sample size of less than 50, or the coefficient of variation of the estimate or its complement is larger than 30%.

[c] Includes Asian, American Indian or Alaska Native, Native Hawaiian, Guamanian or Chamorro, Samoan, other Pacific Islander, and respondents who selected non-Hispanic multiple races.

[d] Current grade level was asked of youth who went to school in the past 12 months. Respondents who selected 5th grade and lower, college, vocational, or technical school, not enrolled, home-schooled, and ungraded were categorized as other (n = 37; data not shown).

[e] Grades were reported by youths' parents or guardians and reflect performance at school in the past 12 months. Excludes ungraded school.

[f] Combusted tobacco products included cigarettes, cigars, pipes, hookah, bidis, and kreteks.

[g] Cigarettes smoked per day was asked of current cigarette smokers.

[h] Non-cigarette combusted tobacco products included cigars, pipes, hookah, bidis, and kreteks.

[i] Non-combusted tobacco products included smokeless tobacco, snus, and dissolvables.

[j] Exclusive ENDS use included respondents who only used ENDS in the past 30 days, not any other tobacco products (combusted or non-combusted).

[k] Current use of any tobacco was reported by youths' parents or guardians, and excludes those who already completed an adult interview and have already completed a parent interview for another youth. Includes any tobacco use from parents in the past 30 days, including cigarettes, traditional cigars, cigarillos, filtered cigars, pipe, hookah, snus, smokeless tobacco, ENDS, and dissolvable tobacco.

* $p < 0.05$, ** $p < 0.01$, *** $p < 0.001$.

Abbreviations: CI = confidence interval; ENDS = electronic nicotine delivery system; OR = unadjusted odds ratio; PATH = Population Assessment of Tobacco and Health; REF = reference level.

Table S3. Characteristics and Patterns of ENDS Use Among Youth Current ENDS Users by Device Type, PATH Study Wave 4.5.[a]

	Overall (n = 724)	Closed Systems (n = 485)	Open Systems (n = 239)	OR[b]	95% CI
	Weighted % (95% CI)	Weighted % (95% CI)	Weighted % (95% CI)		
**Regular brand of ENDS[c]**					
Yes	22.4 (18.9, 26.3)	26.5 (22.5, 31.0)	13.5 (9.2, 19.4)	**2.40*****	(1.48, 3.89)
No	58.6 (54.7, 62.4)	54.7 (49.6, 59.8)	66.9 (60.3, 72.9)	REF	REF
Don’t know	19.0 (16.1, 22.3)	18.8 (15.2, 22.9)	19.6 (15.0, 25.1)	1.17	(0.76, 1.81)
**ENDS device type[d]**				-	-
Disposable	2.5 (1.6, 3.9)	3.6 (2.3, 5.7)	-		
Non-refillable cartridge	15.8 (12.6, 19.6)	23.1 (18.8, 28.0)	-		
Refillable	81.3 (77.6, 84.6)	72.7 (67.9, 76.9)	100.00		
Unknown	0.4 (0.1, 1.2)†	0.6 (0.2, 1.8)†	-		
**Rechargeable[e]**				-	-
Yes	95.4 (93.6, 96.7)	93.3 (90.7, 95.2)	100.0		
No	4.6 (3.3, 6.4)	6.7 (4.8, 9.3)	-		
**Uses cartridges[e]**				-	-
Yes	64.5 (59.8, 68.9)	94.5 (92.1, 96.2)	-		
No	35.5 (31.1, 40.2)	5.5 (3.8, 7.9)	100.0		
**Refillable with e-liquid[e]**				-	-
Yes	81.4 (77.7, 84.6)	72.8 (68.0, 77.0)	100.0		
No	18.6 (15.4, 22.3)	27.2 (23.0, 32.0)	-		
**Ever used ENDS fairly regularly[f]**	52.7 (48.5, 56.7)	50.5 (45.6, 55.3)	57.4 (51.0, 63.5)	0.76	(0.57, 1.01)
**Time since first started using ENDS fairly regularly[g]**					
0 years	28.5 (23.7, 33.8)	29.4 (23.0, 36.7)	26.8 (20.0, 34.8)	REF	REF

1 year	37.8 (32.1, 43.8)	37.4 (31.6, 43.5)	38.6 (28.3, 50.0)	0.88	(0.48, 1.64)
2 – 3 years	24.1 (20.1, 28.6)	21.4 (16.8, 27.0)	29.0 (21.3, 38.2)	0.67	(0.39, 1.18)
>3 years	9.7 (7.0, 13.2)	11.8 (8.0, 17.1)	5.6 (2.7, 11.2)†	1.92	(0.64, 5.74)
**Age first used ENDS regularly**[g]					
<13 years	9.8 (7.0, 13.7)	11.9 (8.1, 17.3)	5.9 (3.0, 11.2)†	REF	REF
≥13 years	90.2 (86.3, 93.0)	88.1 (82.7, 91.9)	94.1 (88.8, 97.0)	0.46	(0.20, 1.08)
**Frequency of current ENDS use**					
1-19 days	76.1 (72.5, 79.3)	78.2 (74.1, 81.8)	71.6 (65.5, 77.0)	REF	REF
20-30 days	23.9 (20.7, 27.5)	21.8 (18.2, 25.9)	28.4 (23.0, 34.5)	**0.70**[*]	(0.50, 0.98)
**Believed ENDS contains nicotine**					
Yes	62.3 (57.8. 66.6)	67.9 (62.2, 73.0)	50.5 (42.2, 58.8)	**2.90**[***]	(1.74, 4.82)
No	24.5 (20.9, 28.5)	17.9 (13.9, 22.8)	38.6 (31.7, 46.1)	REF	REF
Don't know	13.1 (10.8, 15.9)	14.2 (11.4, 17.6)	10.8 (7.5, 15.5)	**2.83**[***]	(1.69, 4.73)
**Flavors used in the past 30 days (choose all that apply)**[h]					
Tobacco flavor	11.3 (9.2, 13.9)	14.7 (11.7, 18.3)	4.1 (2.1, 7.8)†	**4.04**[**]	(1.79, 9.12)
Mint or menthol	44.5 (40.4, 48.8)	53.3 (48.3, 58.2)	25.9 (20.3, 32.5)	**3.26**[***]	(2.23, 4.76)
Clove or spice	2.0 (1.2, 3.4)	2.5 (1.4, 4.3)	1.1 (0.4, 3.6)†	2.19	(0.44, 10.88)
Fruit	78.1 (74.5, 81.4)	77.0 (71.9, 81.3)	80.7 (74.3, 85.8)	0.80	(0.49, 1.32)
Chocolate	3.9 (2.6, 5.8)	4.6 (2.9, 7.3)	2.3 (1.0, 5.2)†	2.05	(0.68, 6.19)
An alcoholic drink	2.2 (1.4, 3.6)	2.2 (1.2, 4.0)†	2.2 (0.8, 6.2)†	1.00	(0.21, 4.83)
A non-alcoholic drink	8.9 (6.7, 11.7)	8.1 (5.5, 11.6)	10.6 (7.2, 15.5)	0.74	(0.40, 1.35)
Candy, desserts, or other sweets	42.0 (38.1, 46.0)	37.9 (33.1, 42.9)	50.8 (44.5, 57.2)	**0.59**[**]	(0.43, 0.81)
Some other flavor	3.7 (2.4, 5.6)	3.3 (1.9, 5.6)	4.5 (2.5, 7.9)	0.73	(0.33, 1.65)
**Reasons for use**					
They are affordable	44.7 (41.1, 48.4)	43.7 (39.1, 48.4)	47.0 (40.1, 54.1)	0.87	(0.61, 1.26)
ENDS can be used in places where smoking cigarettes is not allowed	44.8 (40.8, 49.0)	46.0 (41.1, 50.9)	42.4 (35.8, 49.4)	1.16	(0.83, 1.62)

They might be less harmful to me than smoking cigarettes	69.0 (65.2, 72.6)	68.6 (63.6, 73.3)	69.9 (63.7, 75.3)	0.95	(0.66, 1.36)
They might be less harmful to people around me than cigarettes	66.0 (61.8, 70.0)	63.3 (58.0, 68.3)	71.8 (65.8, 77.2)	**0.68***	(0.48, 0.96)
E-liquid comes in flavors I like	75.2 (71.6, 78.5)	73.1 (68.2, 77.4)	79.9 (74.4, 84.5)	**0.68***	(0.47, 0.98)
Using ENDS helps people quit smoking cigarettes[i]	50.0 (43.9, 56.1)	46.7 (39.4, 54.2)	57.2 (46.4, 67.3)	0.66	(0.38, 1.13)
ENDS don't smell	46.4 (42.7, 50.0)	47.8 (43.0, 52.6)	43.3 (37.1, 49.6)	1.20	(0.84, 1.71)
Using ENDS feels like smoking a regular cigarette	13.8 (11.2, 16.9)	13.0 (10.0, 16.6)	15.6 (10.9, 21.7)	0.81	(0.49, 1.33)
ENDS don't bother non-tobacco users	39.8 (36.2, 43.5)	40.4 (35.7, 45.1)	38.7 (32.7, 45.0)	1.07	(0.77, 1.50)

[a] Analyses are limited to youth not-light current ENDS users. Current ENDS use is any ENDS use in the past 30 days. Not-light current ENDS users used ENDS more than once in their lifetime. For device type – Closed systems are devices that are not rechargeable, or devices that are rechargeable and use cartridges; open systems are devices that are rechargeable, do not use cartridges, and are refillable.

[b] The unadjusted odds ratio is the odds of closed vs. open system use for each level of a variable compared to the reference level for that variable (noted explicitly as REF or implicitly as "no" for binary yes/no variables).

[†] Estimate should be interpreted with caution because it has low precision. It is based on a denominator sample size of less than 50, or the coefficient of variation of the estimate or its complement is larger than 30%.

[c] Regular brand of ENDS was asked of not-light current ENDS users.

[d] ENDS device type was based on responses to the device type questions and only includes non-light current ENDS users. This variable (X04R_Y_EDEVICETYPE_CAT4) is in the RUF extension. Disposable was devices that are not rechargeable, don't use a tank system, don't use cartridges, and not refillable; non-refillable cartridge was devices that use cartridges and are not refillable; refillable was devices that are refillable; and unknown was anything else (including don't know or refused).

[e] A series of binary questions were asked of not-light current ENDS users to categorize the ENDS device they used most often: 1) Is it rechargeable? 2) Does it use cartridges? 3) Can you refill it with "e-liquid"?

[f] Ever use of ENDS fairly regularly imputed values from previous waves (YE/YV1100).

[g] Age at first regular use was asked of regular ENDS users only and values from previous waves were imputed. Time since first use of an e-cigarette was calculated by subtracting YE/YV1007 (how old were you when you first started fairly regular use of ENDS) from current age at Wave 4.5.

[h] Respondents could choose all that apply, so responses don't add up to 100%. For the unadjusted odds ratio, each flavor category was treated like a binary yes/no variable; for each flavor category, the unadjusted odds ratio is the odds of closed vs. open system use for past 30-day use of that flavor compared to the reference level of no past 30-day use of that flavor.

[i] "Using ENDS helps people quit smoking cigarettes" was asked of current ENDS users who have also smoked cigarettes in the past year.

* $p < 0.05$, ** $p < 0.01$, *** $p < 0.001$.

Abbreviations: CI = confidence interval; ENDS = electronic nicotine delivery system; OR = unadjusted odds ratio; PATH = Population Assessment of Tobacco and Health; REF = reference level.

Table S4. Characteristics and Patterns of ENDS Use Among Youth Current ENDS Users by Device Type, PATH Study Wave 5.[a]

	Overall (n = 940)	Closed Systems (n = 549)	Open Systems (n = 391)	OR[b]	95% CI
	Weighted % (95% CI)	Weighted % (95% CI)	Weighted % (95% CI)		
**Regular brand of ENDS[c]**					
Yes	31.9 (28.8, 35.3)	42.6 (38.2, 47.0)	15.7 (12.5, 19.7)	**4.49*****	(3.15, 6.39)
No	51.5 (48.0, 54.9)	40.8 (36.2, 45.5)	67.6 (62.0, 72.8)	REF	REF
Don't know	16.6 (14.0, 19.6)	16.6 (13.4, 20.5)	16.6 (12.6, 21.7)	**1.66***	(1.04, 2.64)
**ENDS device type[d]**				-	-
A disposable device	4.3 (3.1, 6.0)	7.1 (5.1, 9.9)	-		
A device that uses replaceable prefilled cartridges	56.1 (52.4, 59.9)	92.9 (90.1, 94.9)	-		
A device with a tank that you refill with liquids	33.3 (30.0, 36.7)	-	84.1 (79.8, 87.6)		
A mod system	6.3 (4.8, 8.1)	-	15.9 (12.4, 20.2)		
**Ever used ENDS fairly regularly[e]**	59.9 (56.5, 63.3)	59.1 (54.4, 63.7)	61.2 (56.3, 65.9)	0.92	(0.70, 1.20)
**Time since first started using ENDS fairly regularly[f]**					
0 years	26.2 (22.1, 30.7)	26.1 (20.7, 32.3)	26.3 (20.5, 33.2)	REF	REF
1 year	38.3 (33.8, 43.1)	37.6 (32.0, 43.7)	39.4 (32.8, 46.4)	0.96	(0.59, 1.56)
2 – 3 years	27.4 (23.5, 31.8)	29.3 (24.6, 34.4)	24.7 (18.1, 32.7)	1.20	(0.67, 2.14)
>3 years	8.0 (6.0, 10.7)	7.0 (4.5, 10.8)	9.6 (6.5, 14.0)	0.74	(0.36, 1.52)
**Age first used ENDS regularly[f]**					
<13 years	10.6 (8.2, 13.5)	10.0 (7.1, 14.0)	11.4 (7.9, 16.1)	REF	REF
≥13 years	89.4 (86.5, 91.8)	90.0 (86.0, 92.9)	88.6 (83.9, 92.1)	1.15	(0.66, 2.02)
**Frequency of current ENDS use**					
1-19 days	69.8 (65.8, 73.5)	69.4 (64.7, 73.6)	70.5 (65.2, 75.3)	REF	REF
20-30 days	30.2 (26.5, 34.2)	30.6 (26.4, 35.3)	29.5 (24.7, 34.8)	1.06	(0.80, 1.39)

**Believed ENDS contains nicotine**					
Yes	75.0 (71.2, 78.5)	81.2 (76.7, 84.9)	65.6 (60.6, 70.4)	**3.21*****	(2.23, 4.62)
No	14.9 (12.2, 18.2)	9.2 (6.7, 12.4)	23.8 (19.5, 28.6)	REF	REF
Don't know	10.0 (7.6, 13.2)	9.7 (6.8, 13.7)	10.6 (7.6, 14.6)	**2.37****	(1.34, 4.20)
**Flavors used in the past 30 days (choose all that apply)**[g]					
Tobacco flavor	9.9 (8.0, 12.2)	11.6 (8.9, 15.1)	7.3 (5.1, 10.4)	**1.67***	(1.04, 2.66)
Mint or menthol	57.3 (53.2, 61.4)	65.4 (60.7, 69.8)	45.1 (39.2, 51.0)	**2.30*****	(1.71, 3.09)
Clove or spice	1.6 (1.0, 2.7)	1.6 (0.8, 3.2)†	1.5 (0.7, 3.3)†	1.08	(0.37, 3.13)
Fruit	69.3 (65.9, 72.6)	64.1 (59.0, 68.9)	77.4 (72.9, 81.3)	**0.52*****	(0.37, 0.73)
Chocolate	3.0 (1.9, 4.8)	3.1 (1.8, 5.2)	2.9 (1.6, 5.4)†	1.06	(0.51, 2.19)
An alcoholic drink	2.4 (1.5, 4.1)	2.4 (1.3, 4.3)†	2.6 (1.3, 5.0)†	0.92	(0.37, 2.27)
A non-alcoholic drink	6.7 (5.1, 8.9)	3.8 (2.5, 5.9)	11.2 (8.2, 15.1)	**0.32*****	(0.18, 0.55)
Candy, desserts, or other sweets	34.0 (30.3, 38.0)	25.5 (21.2, 30.3)	47.0 (41.5, 52.7)	**0.39*****	(0.28, 0.53)
Some other flavor	1.9 (1.1, 3.0)	1.6 (0.8, 3.1)†	2.2 (1.1, 4.5)†	0.71	(0.25, 2.02)
**Flavor used most often in the past 30 days**[h]					
Tobacco flavor	2.6 (1.7, 3.9)	2.7 (1.5, 4.9)†	2.3 (1.2, 4.6)†	0.50	(0.15, 1.60)
Mint or menthol	32.6 (29.4, 36.0)	42.1 (37.7, 46.7)	18.1 (14.6, 22.3)	REF	REF
Fruit	47.0 (43.8, 50.2)	41.6 (37.2, 46.1)	55.2 (50.2, 60.1)	**0.32*****	(0.23, 0.46)
Candy, desserts, or other sweets	13.6 (11.5, 16.0)	10.3 (8.0, 13.3)	18.4 (14.8, 22.7)	**0.24*****	(0.15, 0.39)
Other flavor	4.3 (3.0, 6.2)	3.3 (1.9, 5.4)	5.9 (3.8, 9.0)	**0.24*****	(0.12, 0.49)
**Reasons for use**					
They are affordable	41.2 (37.6, 44.8)	36.7 (31.9, 41.8)	48.0 (42.2, 53.8)	**0.63****	(0.45, 0.88)
ENDS can be used in places where smoking cigarettes is not allowed	43.7 (40.1, 47.3)	44.1 (39.2, 49.1)	43.0 (38.2, 48.0)	1.04	(0.79, 1.38)
They might be less harmful to me than smoking cigarettes	60.8 (57.7, 63.9)	58.3 (53.8, 62.6)	64.7 (59.8, 69.3)	0.76	(0.57, 1.02)
They might be less harmful to people around me than cigarettes	62.1 (58.8, 65.3)	59.2 (54.2, 64.0)	66.6 (61.2, 71.6)	0.73	(0.52, 1.02)

E-liquid comes in flavors I like	69.4 (66.2, 72.4)	65.3 (61.0, 69.3)	75.6 (70.8, 79.8)	**0.61****	(0.45, 0.82)
Using ENDS helps people quit smoking cigarettes[i]	51.5 (45.9, 57.0)	52.2 (44.2, 60.1)	50.5 (42.2, 58.7)	1.07	(0.66, 1.75)
ENDS don't smell	44.0 (40.9, 47.2)	44.5 (40.3, 48.9)	43.3 (37.7, 49.0)	1.05	(0.76, 1.45)
Using ENDS feels like smoking a regular cigarette	15.4 (12.9, 18.3)	16.0 (12.9, 19.8)	14.5 (11.4, 18.4)	1.13	(0.80, 1.58)
ENDS don't bother non-tobacco users	37.4 (34.0, 41.0)	36.1 (31.5, 41.0)	39.4 (34.4, 44.7)	0.87	(0.64, 1.18)

[a] Analyses are limited to youth not-light current ENDS users. Current ENDS use is any ENDS use in the past 30 days. Not-light current ENDS users used ENDS more than once in their lifetime. For device type – Closed systems are disposable e-cigarettes or e-cigarettes that uses pre-filled pods or cartridges; open systems are e-cigarettes with a refillable tank or mod systems. Device type categorization for Wave 5 includes not-light current ENDS users only (i.e., the primary analytic population).

[b] The unadjusted odds ratio is the odds of closed vs. open system use for each level of a variable compared to the reference level for that variable (noted explicitly as REF or implicitly as "no" for binary yes/no variables).

[†] Estimate should be interpreted with caution because it has low precision. It is based on a denominator sample size of less than 50, or the coefficient of variation of the estimate or its complement is larger than 30%.

[c] Regular brand of ENDS was asked of not-light current ENDS users.

[d] ENDS device type was based on responses to the device type question (R05_YV9001) - type of ENDS used most often. Not-light current ENDS users who selected "something else" (n = 14) were excluded from device type categorization.

[e] Ever use of ENDS fairly regularly imputed values from previous waves (YE/YV1100).

[f] Age at first regular use was asked of regular ENDS users only and values from previous waves were imputed. Time since first use of an e-cigarette was calculated by subtracting YE/YV1007 (how old were you when you first started fairly regular use of ENDS) from current age at Wave 5.

[g] Respondents could choose all that apply, so responses don't add up to 100%. For the unadjusted odds ratio, each flavor category was treated like a binary yes/no variable; for each flavor category, the unadjusted odds ratio is the odds of closed vs. open system use for past 30-day use of that flavor compared to the reference level of no past 30-day use of that flavor.

[h] Flavor used most often in the past 30 days was asked of current ENDS users who used 2 or more flavors in the past 30 days or did not know or refused to report which flavors they used in the past 30 days. Responses for current ENDS users who used 1 flavor in the past 30 days were imputed based on their previous flavor response (n = 438). Other flavor included clove or spice, chocolate, an alcoholic drink, a non-alcoholic drink, or some other flavor.

[i] "Using ENDS helps people quit smoking cigarettes" was asked of current ENDS users who have also smoked cigarettes in the past year.

* p <0.05, ** p <0.01, *** p <0.001.

Abbreviations: CI = confidence interval; ENDS = electronic nicotine delivery system; OR = unadjusted odds ratio; PATH = Population Assessment of Tobacco and Health; REF = reference level.

Table S5. Demographic and Tobacco Use Characteristics of Youth Current ENDS Users by Device Type (Not-Light and Very-Light ENDS Users), PATH Study Wave 5.[a]

	Closed Systems (n = 611)	Open Systems (n = 430)	OR[b]	95% CI
	Weighted % (95% CI)	Weighted % (95% CI)		
**Sex**				
Male	49.8 (45.4, 54.2)	51.5 (46.2, 56.8)	REF	REF
Female	50.2 (45.8, 54.6)	48.5 (43.2, 53.8)	1.07	(0.78, 1.48)
**Age (years)**				
12-13	3.1 (1.9, 5.3)	6.0 (4.2, 8.7)	REF	REF
14-15	30.7 (26.8, 35.0)	33.0 (27.7, 38.9)	1.79	(0.85, 3.75)
16-17	66.1 (61.6, 70.4)	60.9 (55.4, 66.2)	**2.08***	(1.01, 4.29)
**Race/ethnicity**				
White, non-Hispanic	69.3 (65.2, 73.2)	66.8 (62.1, 71.2)	REF	REF
Black, non-Hispanic	4.4 (2.9, 6.8)	6.0 (4.0, 8.9)	0.71	(0.37, 1.38)
Other, non-Hispanic[c]	8.9 (6.7, 11.7)	6.8 (4.7, 9.7)	1.26	(0.83, 1.92)
Hispanic	17.4 (14.5, 20.6)	20.4 (16.8, 24.6)	0.82	(0.60, 1.12)
**Current grade level**[d]				
6-8th grade	6.2 (4.4, 8.6)	11.4 (8.4, 15.4)	REF	REF
9-12th grade	88.4 (85.4, 90.9)	85.3 (81.0, 88.8)	**1.91***	(1.17, 3.12)
**Grades**[e]				
Mostly A's or A's and B's	54.0 (49.6, 58.3)	43.5 (38.5, 48.6)	REF	REF
Mostly B's or B's and C's	27.5 (23.8, 31.5)	34.7 (30.1, 39.7)	**0.64****	(0.47, 0.87)
Mostly C's or C's and D's	13.3 (10.6, 16.6)	15.1 (11.7, 19.4)	0.71	(0.47, 1.07)
Mostly D's, D's and F's, or mostly F's	5.2 (3.7, 7.3)	6.7 (4.5, 9.9)	0.63	(0.37, 1.07)
**Current use of other combusted products**[f]	19.5 (16.3, 23.2)	23.6 (18.9, 29.0)	0.79	(0.56, 1.10)
**Current use of cigarettes**	15.1 (12.2, 18.6)	20.3 (15.7, 25.9)	0.70	(0.48, 1.02)
**Cigarette smoking status**				
Never smoker	59.9 (55.9, 63.7)	53.6 (48.3, 58.8)	REF	REF
Ever smoker, but not within past month	25.0 (21.7, 28.6)	26.2 (22.3, 30.6)	0.85	(0.64, 1.15)
Current smoker, 1-19 days during past month	12.5 (9.8, 15.9)	14.7 (11.2, 19.2)	0.76	(0.49, 1.18)
Current smoker, 20-30 days during past month	2.6 (1.6, 4.2)	5.4 (3.6, 8.1)	**0.43***	(0.22, 0.85)
**Cigarettes smoked per day (current smokers only)**[g]				
<1	48.7 (38.4, 59.1)	35.2 (25.1, 47.0)	REF	REF
1-5	40.4 (30.7, 50.9)	45.2 (33.6, 57.3)	0.65	(0.32, 1.31)

6+	10.9 (5.6, 20.1)†	19.6 (12.0, 30.3)	0.40	(0.12, 1.34)
**Current use of non-cigarette combusted products[h]**	7.4 (5.3, 10.1)	8.2 (5.9, 11.2)	0.89	(0.57, 1.41)
**Current use of non-combusted products[i]**	4.5 (3.1, 6.5)	3.0 (1.8, 5.1)	1.52	(0.85, 2.71)
**Exclusive ENDS use[j]**	77.0 (73.1, 80.5)	74.7 (69.4, 79.3)	1.13	(0.82, 1.57)
**Parents' current use of any tobacco[k]**	30.4 (26.4, 34.7)	42.1 (36.9, 47.4)	**0.60*****	(0.46, 0.78)

[a] Analyses are limited to youth not-light current ENDS users. Current ENDS use is any ENDS use in the past 30 days. Not-light current ENDS users used ENDS more than once in their lifetime; very-light current ENDS users used ENDS only once in their lifetime. For device type – Closed systems are disposable e-cigarettes or e-cigarettes that uses pre-filled pods or cartridges; open systems are e-cigarettes with a refillable tank or mod systems. Device type categorization for Wave 5 includes not-light and very-light current ENDS users and is presented for comparison. Of the 118 very-light current ENDS users in Wave 5, 39 reported open systems and 62 reported closed systems.

[b] The unadjusted odds ratio is the odds of closed vs. open system use for each level of a variable compared to the reference level for that variable (noted explicitly as REF or implicitly as "no" for binary yes/no variables).

† Estimate should be interpreted with caution because it has low precision. It is based on a denominator sample size of less than 50, or the coefficient of variation of the estimate or its complement is larger than 30%.

[c] Includes Asian, American Indian or Alaska Native, Native Hawaiian, Guamanian or Chamorro, Samoan, other Pacific Islander, and respondents who selected non-Hispanic multiple races.

[d] Current grade level was asked of youth who went to school in the past 12 months. Respondents who selected 5th grade and lower, college, vocational, or technical school, not enrolled, home-schooled, and ungraded were categorized as other (n = 40; data not shown).

[e] Grades were reported by youths' parents or guardians and reflect performance at school in the past 12 months. Excludes ungraded school.

[f] Combusted tobacco products included cigarettes, cigars, pipes, hookah, bidis, and kreteks.

[g] Cigarettes smoked per day was asked of current cigarette smokers.

[h] Non-cigarette combusted tobacco products included cigars, pipes, hookah, bidis, and kreteks.

[i] Non-combusted tobacco products included smokeless tobacco, snus, and dissolvables.

[j] Exclusive ENDS use included respondents who only used ENDS in the past 30 days, not any other tobacco products (combusted or non-combusted).

[k] Current use of any tobacco was reported by youths' parents or guardians, and excludes those who already completed an adult interview and have already completed a parent interview for another youth. Includes any tobacco use from parents in the past 30 days, including cigarettes, traditional cigars, cigarillos, filtered cigars, pipe, hookah, snus, smokeless tobacco, ENDS, and dissolvable tobacco.

* p <0.05, ** p <0.01, *** p <0.001.

Abbreviations: CI = confidence interval; ENDS = electronic nicotine delivery system; OR = unadjusted odds ratio; PATH = Population Assessment of Tobacco and Health; REF = reference level.

Table S6. Characteristics and Patterns of ENDS Use Among Youth Current ENDS Users by Device Type (Not-Light and Very-Light ENDS Users), PATH Study Wave 5.[a]

	Closed Systems (n = 611)	Open Systems (n = 430)	OR[b]	95% CI
	Weighted % (95% CI)	Weighted % (95% CI)		
**Regular brand of ENDS[c]**				
Yes	42.6 (38.2, 47.0)	15.7 (12.5, 19.7)	**4.49**[***]	(3.15, 6.39)
No	40.8 (36.2, 45.5)	67.6 (62.0, 72.8)	REF	REF
Don't know	16.6 (13.4, 20.5)	16.6 (12.6, 21.7)	**1.66**[*]	(1.04, 2.64)
**ENDS device type[d]**			-	-
A disposable device	8.4 (6.3, 11.0)	-		
A device that uses replaceable prefilled cartridges	91.6 (89.0, 93.7)	-		
A device with a tank that you refill with liquids	-	83.9 (79.5, 87.5)		
A mod system	-	16.1 (12.5, 20.5)		
**Ever used ENDS fairly regularly[e]**	54.0 (49.4, 58.6)	56.2 (51.4, 60.8)	0.92	(0.71, 1.19)
**Time since first started using ENDS fairly regularly[f]**				
0 years	26.0 (20.7, 32.2)	26.4 (20.5, 33.3)	REF	REF
1 year	37.8 (32.2, 43.6)	39.0 (32.5, 45.8)	0.98	(0.61, 1.58)
2 – 3 years	29.0 (24.4, 34.1)	25.1 (18.5, 33.2)	1.17	(0.66, 2.07)
>3 years	7.2 (4.9, 10.7)	9.5 (6.4, 13.9)	0.78	(0.39, 1.55)
**Age first used ENDS regularly[f]**				
<13 years	10.3 (7.4, 14.1)	11.3 (7.8, 15.9)	REF	REF
≥13 years	89.7 (85.9, 92.6)	88.7 (84.1, 92.2)	1.11	(0.64, 1.93)
**Frequency of current ENDS use**				
1-19 days	71.8 (67.5, 75.7)	72.6 (67.6, 77.2)	REF	REF
20-30 days	28.2 (24.3, 32.5)	27.4 (22.8, 32.4)	1.04	(0.79, 1.37)
**Believed ENDS contains nicotine**				
Yes	76.3 (71.7, 80.4)	62.0 (56.7, 67.1)	**2.92**[***]	(2.03, 4.22)
No	10.9 (8.3, 14.1)	25.9 (21.4, 30.9)	REF	REF
Don't know	12.8 (9.6, 16.8)	12.1 (9.3, 15.6)	**2.51**[***]	(1.54, 4.10)
**Flavors used in the past 30 days (choose all that apply)[g]**				
Tobacco flavor	11.2 (8.6, 14.4)	7.2 (5.1, 10.1)	**1.62**[*]	(1.04, 2.52)
Mint or menthol	63.2 (58.5, 67.5)	43.0 (37.4, 48.8)	**2.27**[***]	(1.70, 3.04)

Clove or spice	1.6 (0.8, 3.1)†	1.4 (0.6, 3.0)†	1.18	(0.42, 3.31)
Fruit	62.6 (57.5, 67.4)	74.9 (70.1, 79.2)	**0.56****	(0.40, 0.79)
Chocolate	3.0 (1.9, 4.9)	2.9 (1.5, 5.3)†	1.05	(0.54, 2.03)
An alcoholic drink	2.1 (1.1, 3.9)†	2.3 (1.2, 4.6)†	0.92	(0.37, 2.28)
A non-alcoholic drink	3.5 (2.2, 5.4)	10.8 (8.0, 14.5)	**0.30*****	(0.17, 0.51)
Candy, desserts, or other sweets	23.7 (19.8, 28.1)	45.2 (40.0, 50.6)	**0.38*****	(0.27, 0.52)
Some other flavor	1.9 (1.0, 3.5)†	2.0 (1.0, 4.0)†	0.94	(0.35, 2.53)
**Flavor used most often in the past 30 days[h]**				
Tobacco flavor	3.0 (1.8, 5.0)	2.5 (1.3, 4.8)†	0.51	(0.19, 1.40)
Mint or menthol	41.8 (37.6, 46.1)	17.9 (14.4, 22.1)	REF	REF
Fruit	41.5 (37.4, 45.7)	54.8 (50.1, 59.5)	**0.32*****	(0.23, 0.45)
Candy, desserts, or other sweets	9.9 (7.7, 12.7)	18.7 (15.0, 23.0)	**0.23*****	(0.14, 0.38)
Other flavor	3.8 (2.4, 5.9)	6.0 (3.8, 9.5)	**0.27*****	(0.14, 0.53)
**Reasons for use**				
They are affordable	35.2 (30.7, 40.0)	45.9 (40.1, 51.9)	**0.64****	(0.46, 0.90)
ENDS can be used in places where smoking cigarettes is not allowed	41.6 (36.9, 46.4)	40.1 (35.4, 45.0)	1.07	(0.80, 1.41)
They might be less harmful to me than smoking cigarettes	55.8 (51.7, 59.7)	63.3 (58.7, 67.8)	**0.73***	(0.56, 0.96)
They might be less harmful to people around me than cigarettes	56.4 (51.8, 61.0)	65.6 (60.5, 70.4)	**0.68***	(0.50, 0.92)
E-liquid comes in flavors I like	61.8 (57.5, 65.9)	73.7 (69.1, 77.9)	**0.58*****	(0.44, 0.77)
Using ENDS helps people quit smoking cigarettes[i]	52.2 (44.3, 59.9)	50.5 (42.1, 58.8)	1.07	(0.66, 1.75)
ENDS don't smell	42.6 (38.6, 46.7)	41.7 (36.6, 47.1)	1.04	(0.77, 1.39)
Using ENDS feels like smoking a regular cigarette	15.8 (12.8, 19.3)	14.4 (11.4, 18.0)	1.12	(0.81, 1.54)
ENDS don't bother non-tobacco users	35.3 (30.7, 40.2)	37.5 (32.5, 42.7)	0.91	(0.67, 1.24)
**Very-light current ENDS use[j]**	9.5 (7.4, 12.2)	9.2 (6.7, 12.6)	1.04	(0.65, 1.67)

[a] Current ENDS use is any ENDS use in the past 30 days. Not-light current ENDS users used ENDS more than once in their lifetime; very-light current ENDS users used ENDS only once in their lifetime. For device type – Closed systems are disposable e-cigarettes or e-cigarettes that uses pre-filled pods or cartridges; open systems are e-cigarettes with a refillable tank or mod systems. Device type categorization for Wave 5 includes not-light and very-light current ENDS users and is presented for comparison. Of the 118 very-light current ENDS users in Wave 5, 39 reported open systems and 62 reported closed systems.

[b] The unadjusted odds ratio is the odds of closed vs. open system use for each level of a variable compared to the reference level for that variable (noted explicitly as REF or implicitly as "no" for binary yes/no variables).

[†] Estimate should be interpreted with caution because it has low precision. It is based on a denominator sample size of less than 50, or the coefficient of variation of the estimate or its complement is larger than 30%.

[c] Regular brand of ENDS was asked of not-light current ENDS users.

[d] ENDS device type was based on responses to the device type question (R05_YV9001) - type of ENDS used most often. Current ENDS users who selected "something else" (n = 20) were excluded from device type categorization.

[e] Ever use of ENDS fairly regularly imputed values from previous waves (YE/YV1100).

[f] Age at first regular use was asked of regular ENDS users only and values from previous waves were imputed. Time since first use of an e-cigarette was calculated by subtracting YE/YV1007 (how old were you when you first started fairly regular use of ENDS) from current age at Wave 5.

[g] Respondents could choose all that apply, so responses don't add up to 100%. For the unadjusted odds ratio, each flavor category was treated like a binary yes/no variable; for each flavor category, the unadjusted odds ratio is the odds of closed vs. open system use for past 30-day use of that flavor compared to the reference level of no past 30-day use of that flavor.
[h] Flavor used most often in the past 30 days was asked of current ENDS users who used 2 or more flavors in the past 30 days or did not know or refused to report which flavors they used in the past 30 days. Responses for current ENDS users who used 1 flavor in the past 30 days were imputed based on their previous flavor response (n = 524). Other flavor included clove or spice, chocolate, an alcoholic drink, a non-alcoholic drink, or some other flavor.
[i] "Using ENDS helps people quit smoking cigarettes" was asked of current ENDS users who have also smoked cigarettes in the past year.
[j] Very-light current ENDS users used ENDS only once in their lifetime.
* p <0.05, ** p <0.01, *** p <0.001.
Abbreviations: CI = confidence interval; ENDS = electronic nicotine delivery system; OR = unadjusted odds ratio; PATH = Population Assessment of Tobacco and Health; REF = reference level.

Table S7. Demographic and Tobacco Use Characteristics of Youth Current ENDS Users by Not-Light vs. Very-Light ENDS Use, PATH Study Wave 4.5.[a]

	Not-Light (n = 765)	Very-Light (n = 104)	OR[b]	95% CI
	Weighted % (95% CI)	Weighted % (95% CI)		
**Prevalence**	88.8 (86.5, 90.8)	11.2 (9.2, 13.5)		
**Sex**				
Male	51.4 (47.6, 55.3)	37.1 (28.4, 46.7)	REF	REF
Female	48.6 (44.7, 52.4)	62.9 (53.3, 71.6)	**1.80****	(1.18, 2.74)
**Age (years)**				
12-13	4.5 (3.2, 6.5)	8.0 (3.8, 16.0)†	REF	REF
14-15	31.2 (28.0, 34.6)	37.5 (28.8, 47.1)	0.68	(0.27, 1.73)
16-17	64.3 (60.8, 67.6)	54.5 (44.8, 63.9)	0.48	(0.18, 1.28)
**Race/ethnicity**				
White, non-Hispanic	70.5 (67.1, 73.7)	64.5 (53.5, 74.1)	REF	REF
Black, non-Hispanic	3.6 (2.4, 5.3)	5.3 (2.0, 13.3)†	1.61	(0.44, 5.90)
Other, non-Hispanic[c]	8.0 (6.3, 10.2)	9.1 (4.9, 16.5)†	1.25	(0.56, 2.76)
Hispanic	17.9 (15.3, 20.8)	21.1 (13.5, 31.3)	1.29	(0.73, 2.27)
**Current grade level[d]**				
6-8th grade	5.8 (4.2, 7.9)	10.9 (5.7, 19.8)†	REF	REF
9-12th grade	90.8 (88.0, 93.0)	88.3 (79.2, 93.7)	0.52	(0.22, 1.22)
**Grades[e]**				
Mostly A's or A's and B's	52.3 (48.0, 56.6)	53.0 (42.4, 63.4)	REF	REF
Mostly B's or B's and C's	29.3 (25.9, 32.9)	32.4 (22.5, 44.2)	1.09	(0.63, 1.89)
Mostly C's or C's and D's	13.6 (10.9, 16.7)	11.7 (6.5, 20.0)	0.85	(0.41, 1.77)
Mostly D's, D's and F's, or mostly F's	4.8 (3.5, 6.6)	2.9 (1.0, 8.3)†	0.59	(0.17, 2.09)
**Current use of other combusted products[f]**	25.8 (22.8, 29.2)	15.9 (9.5, 25.4)	**0.54***	(0.30, 1.00)
**Current use of cigarettes**	21.2 (18.3, 24.4)	12.9 (7.2, 22.0)	0.55	(0.28, 1.09)
**Cigarette smoking status**				
Never smoker	53.0 (49.0, 57.0)	71.1 (60.7, 79.7)	REF	REF

Ever smoker, but not within past month	25.9 (22.5, 29.6)	17.0 (10.7, 26.0)	**0.49***	(0.28, 0.86)
Current smoker, 1-30 days during past month[g]	21.1 (18.2, 24.2)	11.9 (6.4, 21.2)†	**0.42***	(0.20, 0.87)
**Cigarettes smoked per day (current smokers only)[h]**				
<1	34.8 (27.8, 42.5)	53.6 (27.5, 78.0)†	REF	REF
1-5	54.0 (45.5, 62.3)	46.4 (22.0, 72.5)†	-	-
6+	11.2 (7.2, 17.0)	0	-	-
**Current use of non-cigarette combusted products[i]**	10.5 (8.5, 12.9)	9.0 (4.1, 18.5)†	0.84	(0.31, 2.29)
**Current use of non-combusted products[j]**	6.3 (4.7, 8.3)	3.2 (0.8, 11.2)†	0.49	(0.07, 3.26)
**Exclusive ENDS use[k]**	71.1 (67.8, 74.2)	82.9 (73.3, 89.5)	**1.97***	(1.08, 3.59)
**Parents' current use of any tobacco[l]**	28.7 (24.8, 33.1)	29.2 (21.0, 39.1)	1.03	(0.63, 1.67)

[a] Current ENDS use is any ENDS use in the past 30 days. Not-light current ENDS users used ENDS more than once in their lifetime; very-light current ENDS users used ENDS only once in their lifetime. In comparison with very-light current ENDS users, the mean number of days that not-light current ENDS users used an ENP in the past 30 days was 9.7 days (median = 4.3, range = 29.0; p <0.001 for difference in means).
[b] The unadjusted odds ratio is the odds of very-light vs. not-light current ENDS use for each level of a variable compared to the reference level for that variable (noted explicitly as REF or implicitly as "no" for binary yes/no variables).
† Estimate should be interpreted with caution because it has low precision. It is based on a denominator sample size of less than 50, or the coefficient of variation of the estimate or its complement is larger than 30%.
[c] Includes Asian, American Indian or Alaska Native, Native Hawaiian, Guamanian or Chamorro, Samoan, other Pacific Islander, and respondents who selected non-Hispanic multiple races.
[d] Current grade level was asked of youth who went to school in the past 12 months. A total of 45 respondents were missing in the full sample of all current ENDS users. Respondents who selected 5th grade and lower, college, vocational, or technical school, not enrolled, home-schooled, and ungraded were categorized as other (n = 27; data not shown).
[e] Grades were reported by youths' parents or guardians and reflect performance at school in the past 12 months. Excludes ungraded school.
[f] Combusted tobacco products included cigarettes, cigars, pipes, hookah, bidis, and kreteks.
[g] Due to an unweighted sample size <3, current smoker, 1-19 days and current smoker, 20-30 days were combined as current smoker, 1-30 days during past month.
[h] Cigarettes smoked per day was asked of current cigarette smokers. A total of 692 respondents were missing in the full sample of all current ENDS users (only 1 missing from current cigarette smokers).
[i] Non-cigarette combusted tobacco products included cigars, pipes, hookah, bidis, and kreteks.
[j] Non-combusted tobacco products included smokeless tobacco, snus, and dissolvables.
[k] Exclusive ENDS use included respondents who only used ENDS in the past 30 days, not any other tobacco products (combusted or non-combusted).
[l] Current use of any tobacco was reported by youths' parents or guardians, and excludes those who already completed a parent interview for another youth. Includes any tobacco use from parents in the past 30 days, including cigarettes, traditional cigars, cigarillos, filtered cigars, pipe, hookah, snus, smokeless tobacco, ENDS, and dissolvable tobacco.
* p <0.05, ** p <0.01, *** p <0.001.
Abbreviations: CI = confidence interval; ENDS = electronic nicotine delivery system; ENP = electronic nicotine product; OR = unadjusted odds ratio; PATH = Population Assessment of Tobacco and Health; REF = reference level.

Table S8. Demographic and Tobacco Use Characteristics of Youth Current ENDS Users by Not-Light vs. Very-Light ENDS Use, PATH Study Wave 5.[a]

	Not-Light (n = 965)	Very-Light (n = 118)	OR[b]	95% CI
	**Weighted % (95% CI)**	**Weighted % (95% CI)**		
**Prevalence**	89.6 (87.7, 91.3)	10.4 (8.7, 12.3)		
**Sex**				
Male	50.8 (47.6, 53.9)	46.5 (37.1, 56.2)	REF	REF
Female	49.2 (46.1, 52.4)	53.5 (43.8, 62.9)	1.19	(0.76, 1.85)
**Age (years)**				
12-13	4.3 (3.2, 5.8)	7.9 (3.7, 16.1)†	REF	REF
14-15	30.4 (27.4, 33.6)	44.5 (35.0, 54.5)	0.80	(0.28, 2.28)
16-17	65.3 (62.0, 68.5)	47.5 (37.9, 57.3)	0.40	(0.14, 1.13)
**Race/ethnicity**				
White, non-Hispanic	68.8 (65.5, 71.9)	54.5 (45.4, 63.2)	REF	REF
Black, non-Hispanic	5.2 (3.9, 6.9)	3.8 (1.5, 9.0)†	0.92	(0.30, 2.87)
Other, non-Hispanic[c]	8.2 (6.6, 10.2)	11.1 (6.6, 18.2)	1.71	(0.95, 3.09)
Hispanic	17.8 (15.5, 20.4)	30.6 (23.0, 39.5)	**2.17*****	(1.41, 3.32)
**Current grade level[d]**				
6-8th grade	7.9 (6.1, 10.1)	15.9 (9.4, 25.7)	REF	REF
9-12th grade	87.5 (85.0, 89.6)	81.6 (71.8, 88.6)	**0.46***	(0.22, 0.96)
**Grades[e]**				
Mostly A's or A's and B's	49.2 (45.8, 52.6)	48.8 (39.6, 58.1)	REF	REF
Mostly B's or B's and C's	30.2 (27.3, 33.2)	34.8 (26.4, 44.2)	1.16	(0.75, 1.80)
Mostly C's or C's and D's	14.6 (12.5, 17.0)	12.0 (6.6, 21.0)†	0.83	(0.40, 1.71)
Mostly D's, D's and F's, or mostly F's	6.0 (4.5, 8.0)	4.3 (1.9, 9.7)†	0.73	(0.33, 1.62)
**Current use of other combusted products[f]**	22.1 (19.1, 25.4)	13.8 (7.9, 23.0)	0.56	(0.28, 1.13)
**Current use of cigarettes**	18.3 (15.4, 21.6)	6.1 (2.4, 14.9)†	0.29	(0.08, 1.02)
**Cigarette smoking status**				
Never smoker	55.3 (52.1, 58.4)	79.7 (71.3, 86.1)	REF	REF
Ever smoker, but not within past month	26.5 (23.8, 29.3)	14.2 (9.0, 21.6)	**0.37*****	(0.22, 0.62)
Current smoker, 1-30 days during past month[g]	18.2 (15.3, 21.5)	6.1 (2.4, 14.9)†	**0.23***	(0.07, 0.80)
**Cigarettes smoked per day (current smokers only)[h]**				
<1	41.4 (34.5, 48.7)	[S]	REF	REF
1-5	44.1 (35.8, 52.7)	[S]	0.69	(0.06, 7.97)
6+	14.5 (10.0, 20.6)	[S]	0.86	(0.04, 18.79)

**Current use of non-cigarette combusted products[i]**	7.7 (6.1, 9.7)	8.2 (4.3, 15.1)†	1.07	(0.49, 2.33)
**Current use of non-combusted products[j]**	4.2 (3.1, 5.7)	0	-	-
**Exclusive ENDS use[k]**	74.9 (71.6, 77.8)	86.1 (76.8, 92.0)	**2.08***	(1.05, 4.13)
**Parents' current use of any tobacco[l]**	36.3 (32.6, 40.1)	22.5 (15.8, 30.9)	**0.51****	(0.33, 0.79)

[a] Current ENDS use is any ENDS use in the past 30 days. Not-light current ENDS users used ENDS more than once in their lifetime; very-light current ENDS users used ENDS only once in their lifetime. In comparison with very-light current ENDS users, the mean number of days that not-light current ENDS users used an ENP in the past 30 days was 11.4 days (median = 5.2, range = 29.0; p <0.001 for difference in means).

[b] The unadjusted odds ratio is the odds of very-light vs. not-light current ENDS use for each level of a variable compared to the reference level for that variable (noted explicitly as REF or implicitly as "no" for binary yes/no variables).

[†] Estimate should be interpreted with caution because it has low precision. It is based on a denominator sample size of less than 50, or the coefficient of variation of the estimate or its complement is larger than 30%.

[c] Includes Asian, American Indian or Alaska Native, Native Hawaiian, Guamanian or Chamorro, Samoan, other Pacific Islander, and respondents who selected non-Hispanic multiple races.

[d] Current grade level was asked of youth who went to school in the past 12 months. Respondents who selected 5th grade and lower, college, vocational, or technical school, not enrolled, home-schooled, and ungraded were categorized as other (n = 40; data not shown).

[e] Grades were reported by youths' parents or guardians and reflect performance at school in the past 12 months. Excludes ungraded school.

[f] Combusted tobacco products included cigarettes, cigars, pipes, hookah, bidis, and kreteks.

[g] Due to an unweighted sample size <3, current smoker, 1-19 days and current smoker, 20-30 days were combined as current smoker, 1-30 days during past month.

[h] Cigarettes smoked per day was asked of current cigarette smokers. A total of 896 respondents were missing in the full sample of all current ENDS users (none missing from current cigarette smokers).

[S] Unweighted sample size is <3 (non-zero) and values are suppressed.

[i] Non-cigarette combusted tobacco products included cigars, pipes, hookah, bidis, and kreteks.

[j] Non-combusted tobacco products included smokeless tobacco, snus, and dissolvables.

[k] Exclusive ENDS use included respondents who only used ENDS in the past 30 days, not any other tobacco products (combusted or non-combusted).

[l] Current use of any tobacco was reported by youths' parents or guardians, and excludes those who already completed an adult interview and have already completed a parent interview for another youth. Includes any tobacco use from parents in the past 30 days, including cigarettes, traditional cigars, cigarillos, filtered cigars, pipe, hookah, snus, smokeless tobacco, ENDS, and dissolvable tobacco.

* p <0.05, ** p <0.01, *** p <0.001.

Abbreviations: CI = confidence interval; ENDS = electronic nicotine delivery system; ENP = electronic nicotine product; OR = unadjusted odds ratio; PATH = Population Assessment of Tobacco and Health; REF = reference level.

Figure S1. Frequency (1-19 vs. 20-30 Days) of Current ENDS Use in Youth Not-Light Current ENDS Users - Overall, PATH Study Waves 4, 4.5, and 5.[a,b]

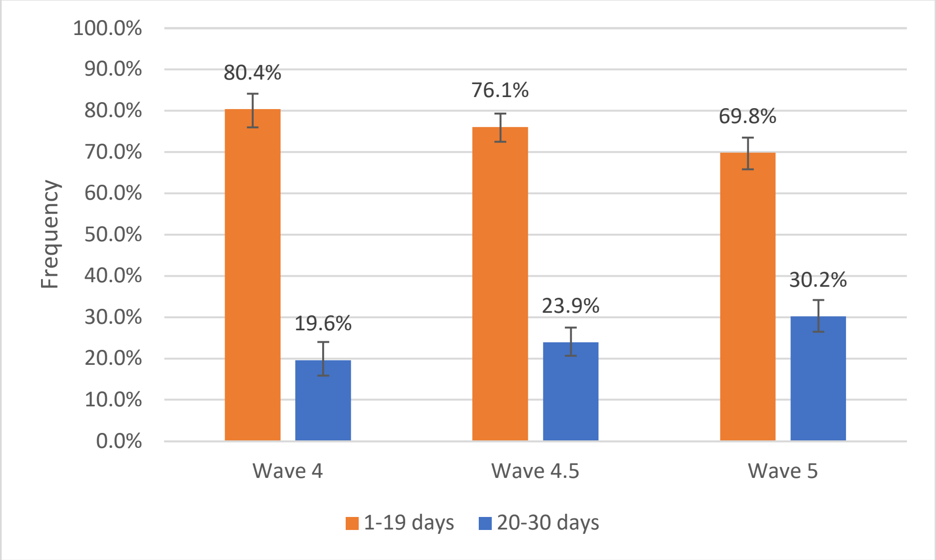

Compared to current ENDS use on 1-19 days, the odds of use on 20-30 days for each one wave increase was 1.34 times (95% CI 1.16, 1.56) the odds of use on 20-30 days at the previous wave ($p<0.001$).[c]

[a] Current ENDS use is any ENDS use in the past 30 days. Not-light current ENDS users used ENDS more than once in their lifetime. For device type – Waves 4 and 4.5: Closed systems are devices that are not rechargeable, or devices that are rechargeable and use cartridges; open systems are devices that are rechargeable, do not use cartridges, and are refillable. Wave 5: Closed systems are disposable e-cigarettes or e-cigarettes that uses pre-filled pods or cartridges; open systems are e-cigarettes with a refillable tank or mod systems. Device type categorization for Wave 5 includes not-light current ENDS users only (i.e., the primary analytic population).

[b] Wave 4 is 2016-2017, Wave 4.5 is 2017-2018, and Wave 5 is 2018-2019. For each wave, percentage estimates of the frequency of current ENDS use are weighted.

[c] Unadjusted logistic regression was used to test for a trend (cross-sectional) in the frequency of current ENDS use (20-30 days compared to 1-19 days [REF]) overall across waves.

Abbreviations: ENDS = Electronic nicotine delivery system; PATH = Population Assessment of Tobacco and Health; REF = reference level.

Figure S2. Frequency (1-19 vs. 20-30 Days) of Current ENDS Use in Youth Not-Light Current ENDS Users – Open Systems, PATH Study Waves 4, 4.5, and 5.[a,b]

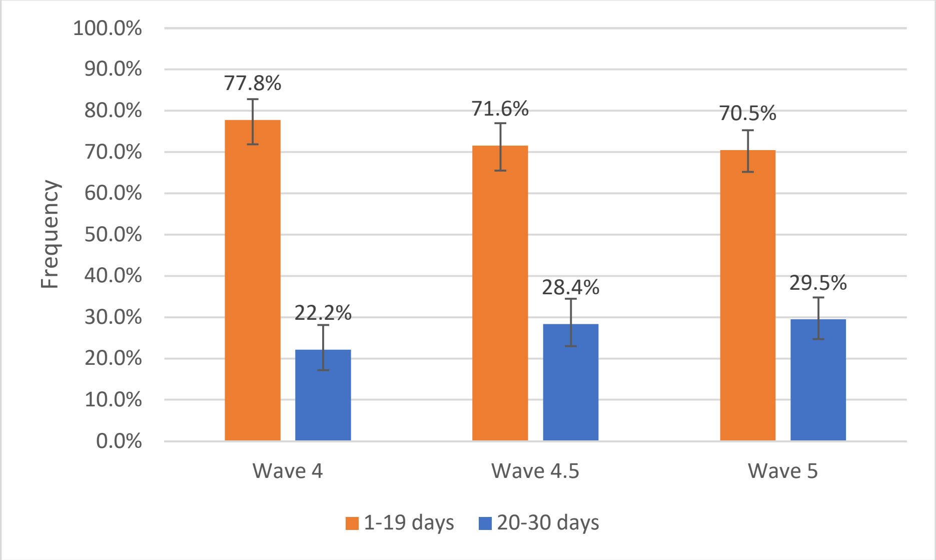

For open systems: Compared to current ENDS use on 1-19 days, the odds of use on 20-30 days for each one wave increase was 1.19 times (95% CI 0.97, 1.46) the odds of use on 20-30 days at the previous wave (p=0.089).[c]

[a] Current ENDS use is any ENDS use in the past 30 days. Not-light current ENDS users used ENDS more than once in their lifetime. For device type – Waves 4 and 4.5: Closed systems are devices that are not rechargeable, or devices that are rechargeable and use cartridges; open systems are devices that are rechargeable, do not use cartridges, and are refillable. Wave 5: Closed systems are disposable e-cigarettes or e-cigarettes that uses pre-filled pods or cartridges; open systems are e-cigarettes with a refillable tank or mod systems. Device type categorization for Wave 5 includes not-light current ENDS users only (i.e., the primary analytic population).

[b] Wave 4 is 2016-2017, Wave 4.5 is 2017-2018, and Wave 5 is 2018-2019. For each wave, percentage estimates of the frequency of current ENDS use are weighted.

[c] Unadjusted logistic regression was used to test for a trend (cross-sectional) in the frequency of current ENDS use (20-30 days compared to 1-19 days [REF]) in open systems across waves.

Abbreviations: ENDS = Electronic nicotine delivery system; PATH = Population Assessment of Tobacco and Health; REF = reference level.

Figure S3. Frequency (1-19 vs. 20-30 Days) of Current ENDS Use in Youth Not-Light Current ENDS Users – Closed Systems, PATH Study Waves 4, 4.5, and 5.[a,b]

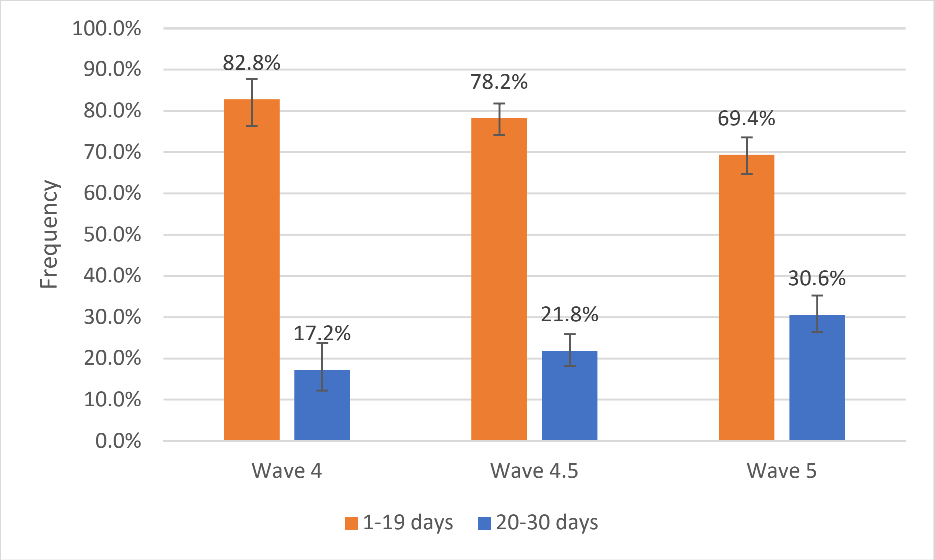

For closed systems: Compared to current ENDS use on 1-19 days, the odds of use on 20-30 days for each one wave increase was 1.49 times (95% CI 1.21, 1.84) the odds of use on 20-30 days at the previous wave (p<0.001).[c]

[a] Current ENDS use is any ENDS use in the past 30 days. Not-light current ENDS users used ENDS more than once in their lifetime. For device type – Waves 4 and 4.5: Closed systems are devices that are not rechargeable, or devices that are rechargeable and use cartridges; open systems are devices that are rechargeable, do not use cartridges, and are refillable. Wave 5: Closed systems are disposable e-cigarettes or e-cigarettes that uses pre-filled pods or cartridges; open systems are e-cigarettes with a refillable tank or mod systems. Device type categorization for Wave 5 includes not-light current ENDS users only (i.e., the primary analytic population).

[b] Wave 4 is 2016-2017, Wave 4.5 is 2017-2018, and Wave 5 is 2018-2019. For each wave, percentage estimates of the frequency of current ENDS use are weighted.

[c] Unadjusted logistic regression was used to test for a trend (cross-sectional) in the frequency of current ENDS use (20-30 days compared to 1-19 days [REF]) in closed systems across waves.

Abbreviations: ENDS = Electronic nicotine delivery system; PATH = Population Assessment of Tobacco and Health; REF = reference level.
